# An Exploratory Comparative Study of the Wechsler Intelligence Scale for Children—Fifth Edition (WISC-V) and the Adaptive Intelligence Diagnosticum 3 (AID 3) in a Sample of Mathematically Highly Gifted Children and Adolescents

**DOI:** 10.3390/jintelligence14040052

**Published:** 2026-03-26

**Authors:** Sophie Alina Schneider, Nina Krüger

**Affiliations:** Department Differential Psychology and Psychological Assessment, Institute of Psychology, University of Hamburg, 20146 Hamburg, Germany

**Keywords:** intelligence test batteries, AID 3, WISC-V, adolescence, children, comparability, assessment of intelligence, high intellectual ability, giftedness

## Abstract

Intelligence test batteries are a common tool in psychological assessment. Their results can have a large impact on an individual’s life, especially for children and adolescents. Despite this, uncertainty remains as to what extent these results are dependent on the test battery used. Two commonly used intelligence test batteries for children and adolescents in German speaking countries are the WISC-V and the AID 3. This study aimed to investigate the degree of comparability between the two test batteries in terms of their resulting scores, subtest content and test profiles in a mathematically gifted sample. A total of 36 children and adolescents (aged *M* = 12.89 years, *SD* = 0.58) completed all subtests of both test batteries. Results revealed that most IQ measures did not differ significantly between the two test batteries for this sample. The correlations of the subtests revealed a structure with four main nodes that was in line with previous factor analytical studies. The standard deviations of the τ-adjusted test scores within test profiles were not significantly different; however, significantly higher ranges were found in the AID 3. Results indicate higher IQ scores on the WISC-V, differential validity for factor structures, and methodological benefits of adaptive testing with the AID 3, particularly in gifted samples. Despite subtest overlaps, composite scores diverge and require individualized interpretation.

## 1. Introduction

Intelligence is widely recognized as one of the most extensively assessed cognitive abilities globally (de Jong, 2023). Over decades of research, intelligence test batteries have evolved into sophisticated tools tailored to specific populations and testing objectives. However, a notable challenge remains: there is no unified terminology, definition, or standardized guideline ensuring comparability of scores across different intelligence assessments (Mickley & Renner, 2019). While the Standards for Educational and Psychological Testing (AERA et al., 2014) provide overarching principles for test validity and fairness, they do not prescribe specific guidelines for score comparability between different intelligence tests, contributing to ongoing challenges in harmonizing assessments. This is striking, considering the magnitude of decisions that are often made (partly) based on intelligence test results derived from a single most available intelligence test out of a range of existing tests. In children, intelligence testing is utilized to inform a range of educational decisions (de Jong, 2023). The authors of intelligence test batteries claim their instruments can aid in identifying impairment of intelligence and learning disorders, in making decisions on interventions and placements, and in finding answers to neuropsychological questions (e.g., Kubinger & Holocher-Benetka, 2023; Wechsler, 2017). However, depending exclusively on IQ scores is inadequate for diagnostic purposes, such as accurately identifying learning disabilities or developmental impairments or even high abilities, as it may overlook other critical factors necessary for precise diagnoses and intervention planning (Krüger et al., 2021). This point becomes especially crucial if different test batteries yield different results and thus result in different diagnostical and educational decisions. This study aims to investigate the magnitude of this issue by empirically comparing the German edition of the Wechsler Intelligence Scale for Children—Fifth Edition (WISC-V; Wechsler, 2017) and the German edition of the Adaptive Intelligence Diagnosticum (AID 3; Kubinger & Holocher-Benetka, 2023), two widely used intelligence test batteries for children in Germany (Bölte et al., 2000).

### 1.1. Intelligence Theories

To contextualize the assessments, it is essential to review the relevant theoretical frameworks underpinning intelligence measurement, particularly as they inform the structure and content of the WISC-V and AID 3.

*Spearman’s g-factor.* Most contemporary scholars agree that a general intelligence factor (g) exists (Spearman, 1904; e.g., Deary et al., 2010), although some debate persists regarding its utility and generalizability, which are sometimes questioned (e.g., Kubinger & Holocher-Benetka, 2023; Süß & Beauducel, 2011). Commonly it is expressed in IQ-values to quantify an individual’s intelligence score essentially as a deviation from the mean of the respective individual’s age group (Preckel & Vock, 2021; Süß & Beauducel, 2011).

*Cattell-Horn-Carroll Theory.* The Cattell-Horn-Carroll theory (CHC theory) developed by McGrew (1997, 2009) is currently the most popular intelligence structure model (Preckel & Vock, 2021). Within the CHC theory, there are three hierarchical levels with the g-factor at the top level, followed by 16 broad abilities and finally specific cognitive abilities at the lowest level (Carroll, 1993; de Jong, 2023). This model affects implicitly or explicitly the advancement of various intelligence test batteries (Mickley & Renner, 2019).

*Giftedness* is commonly understood as markedly superior intellectual ability, often operationalized through IQ scores exceeding 130 or 145 (Gagné, 2004). However, traditional intelligence tests are susceptible to ceiling effects, which can constrain their capacity to differentiate among highly gifted individuals, potentially leading to underestimation of their abilities (Balchin et al., 2009; Hattie & Zierer, 2019). These limitations underscore the importance of employing assessment tools that remain sensitive at the high end of the ability spectrum.

### 1.2. Mathematical Giftedness and Intelligence

While intelligence (IQ) has traditionally been regarded as a key indicator of mathematical giftedness (Kontoyianni et al., 2013; Lohman, 2005; Warne, 2016), this perspective has been subject to ongoing debate. Käpnick et al. (2005) emphasize that relying solely on IQ may be insufficient for defining mathematical giftedness. While intelligence strongly predicts academic performance, particularly in mathematics (Lauermann et al., 2020; B. Roth et al., 2015), using it as the sole measure is inadequate (Hattie & Timperley, 2020; Kießwetter, 1985; Lohman et al., 2008; Nolte, 2011). This is because it fails to capture the multidimensionality of mathematical giftedness and leaves significant unexplained variance, indicating the relevance of additional individual traits (Kießwetter, 1985; Lohman et al., 2008; Nolte, 2011).

In Nolte’s (2011) study, the fluid reasoning subtest from the CFT 20-R (Weiß, 2019) demonstrated limited predictive power for mathematical talent, despite a high number of students with IQs ≥ 130 being identified as gifted. This suggests that additional cognitive and non-cognitive factors contribute to mathematical giftedness, underscoring the importance of integrating multiple assessment approaches.

Recent research (Hattie & Zierer, 2019) confirms this and emphasizes that while IQ tests provide valuable insights into mathematical achievement, they often fail to capture all facets of mathematical giftedness, especially at the upper ability levels where ceiling effects are prevalent. These studies highlight the necessity of multi-method assessments to accurately identify and support gifted learners.

### 1.3. Test Theory

Psychometric principles underlying intelligence testing are rooted in two major frameworks: Classical Test Theory (CTT) and Item Response Theory (IRT). CTT, the traditional approach, models test scores as the sum of individual item responses, assuming that test reliability and validity are consistent across different populations (Gulliksen, 1950). It offers straightforward score interpretation via total or composite scores but faces notable limitations. These include population dependence—meaning that item parameters and scores can vary across groups—and reduced sensitivity at the extremes of ability, which may result in ceiling or floor effects (Krumm et al., 2022; Gonthier et al., 2017). Furthermore, CTT assumes that all items contribute equally and relies on a deterministic scoring approach—assumptions that have been subject to critique.

In contrast, IRT employs a probabilistic framework that models each respondent’s ability based on the likelihood of answering individual items correctly, taking into account item difficulty and discrimination parameters (Lord & Novick, 1968; Rasch, 1960). Rasch’s model, the simplest form of IRT, aims for invariant measurement, where person ability and item difficulty are estimated independently, resulting in a scale that remains stable across different samples (Fischer, 1978; Valek, 2017). IRT-based methods provide more precise measurement at all ability levels, especially at the upper end where ceiling effects are more prevalent, and facilitate the development of adaptive and even tailored assessments that adapt dynamically to an individual’s ability (Gonthier et al., 2017; Kubinger, 2014).

The WISC-V primarily relies on CTT principles, utilizing sum scores and factor analytic validation of its structure (Wechsler, 2017). Conversely, the AID 3 is based on IRT and incorporates extensive adaptive testing algorithms, allowing for efficient, precise assessments across a broad range of ability levels (Kubinger & Holocher-Benetka, 2023). The integration of these two frameworks exemplifies the evolution in psychometric testing—from simple item summation to sophisticated, model-based measurement—enhancing both accuracy and clinical utility.

### 1.4. Test Batteries

In this next section, the two test batteries used in this study will be introduced in terms of their history and content with regard to the methods used in this study. Additionally, the internal validity reported for the WISC-V and the AID 3, respectively, will be considered as a basis for expected correlation structures.

#### 1.4.1. Wechsler Intelligence Scale for Children—Fifth Edition

The WISC-V (Wechsler, 2017) is the most commonly used test battery in German speaking countries to assess cognitive ability in children and adolescents between 6;0 and 16;11 years of age (Bölte et al., 2000; Pisters et al., 2022; Preckel & Vock, 2021). The WISC-V follows the long tradition of the Wechsler scales, more specifically of the WISCs. Wechsler’s intelligence scales have traditionally been associated with Spearman’s *g*-factor theory. However, newer Wechsler scales were adapted to fit the CHC-theory. Among those, there is also the WISC-V (Pisters et al., 2022), even though this was not specifically mentioned in the German manual (Canivez et al., 2021). The overarching theoretical subject of Wechsler scales, including the WISC-V, however, continues to have “clinical usefulness” (Pisters et al., 2022).

*Content, Structure, and Application Area.* The German WISC-V consists of 10 primary and five secondary subtests which can be combined into 11 composite scores, namely, the Full Scale IQ (FSIQ), with five primary and five ancillary indexes. Pisters et al. (2022) reasoned that the five primary indexes are probably conceptualized to represent one factor each on the second stratum of the CHC model even though this was not unambiguously apparent. Individual subtest scores are usually not interpreted in the WISC-V. A translation between the German and English subtest and composite score names, the subtest instructions, and the exact composition of each composite score, can be found in Supplementary Materials S1, S3, and S5, respectively. The WISC-V provides a wide range of possible additional analyses, which, however, will not be relevant for this study and thus not considered further. In the WISC-V, all subtests are tested conventionally (Wechsler, 2017).

*Internal Validity.* Both exploratory (EFA) and confirmatory factor analyses (CFA) are common tools for assessing and reporting internal validity. EFA are often conducted to first explore the factor structure of given variables in the absence of previous studies or robust results. CFA, on the other hand, are often conducted if there is already an assumption about the factor structure, e.g., from previous studies or theoretical considerations (Wechsler, 2017).

As there were specific assumptions about the underlying intelligence model, only CFA results regarding the primary indexes were reported in the WISC-V manual (Wechsler, 2017), even though some researchers argue that test revisions should be treated like new tests and thus perform and report EFAs (Beaujean, 2015). A five-factor model of intelligence was chosen because it combined satisfactory goodness-of-fit statistics and accordance with the theoretical assumptions underlying the test construction (Wechsler, 2017). Ancillary indexes were not considered in the validation process described in the manual (Pisters et al., 2022).

While the WISC is widely used and validated, recent research has indicated inconsistent findings regarding its underlying factor structure. For instance, some studies have employed exploratory factor analysis (EFA) to investigate the dimensionality of the WISC, revealing alternative solutions to the original manuals’ models. Furthermore, confirmatory factor analyses (CFA) in several German and international samples have produced contradicting results, suggesting that the factorial validity of the WISC may not be universally stable (e.g., de Jong, 2023; Canivez et al., 2021; Pauls & Daseking, 2021). These discrepancies highlight the importance of further examining the factorial structure of the WISC, especially in different cultural and linguistic contexts.

In Germany, Pauls and Daseking (2021) conducted a CFA on the German version of the WISC-V and found indications that a four-factor model may fit the data better than the proposed five-factor structure. Similar inconsistencies have been reported in international samples, with some studies supporting alternative solutions such as a three-factor or bifactor model (e.g., Canivez et al., 2021; Pauls & Daseking, 2021). These findings underscore the ongoing debate about the factorial integrity of the WISC and the necessity for further systematic analyses.

#### 1.4.2. Adaptive Intelligence Diagnosticum 3

AID was first published in 1985 (Kubinger & Wurst, 1985), and is currently available in its third edition (Kubinger & Holocher-Benetka, 2023)^1^. It is an intelligence test battery for ages 6;0 to 15;11 and largely follows the WISC-tradition content-wise. Kubinger (e.g., Kubinger, 1983, 1998a, 1998b) found it necessary to publish a new test to assess intelligence in children because of the insufficient psychometric base in the WISC that makes the interpretation of the derived scores questionable. In multiple studies on different WISC editions, he heavily criticized the WISC for problematic scaling and fairness. The construction of the AID was therefore originally mainly aimed at combatting methodological issues found in the WISC and offering a useful alternative (Kubinger, 1983). Thus, the AID 3, as its predecessor, is based on the RM and features adaptive testing (Kubinger, 1983, 2017b).

*Content, Structure, and Application area.* The AID 3 consists of 12 main subtests and five add-on tests, their names in the German and English edition can be viewed in Supplementary Materials S2; their instructions can be found in Supplementary Materials S3. Since the test authors pursue the general goal of providing a basis for the enhancement of abilities in children with various deficits, the importance of interpreting each respective subtest as one cognitive ability is highlighted (Kubinger & Holocher-Benetka, 2023). In contrast to the WISC-V, the AID 3 does not recommend composite scores although two are provided to accommodate institutional needs. The authors’ reasoning for this choice lies in the fact that (1) their factor analyses have not supported a *g*-factor solution, (2) they conceptually do not support a compensation model, and (3) they do not see any practical usefulness for an IQ (Holocher-Ertl et al., 2006, 2008; Kubinger & Holocher-Benetka, 2023). A compensation model of intelligence would postulate the idea that a deficit in one area can be compensated with a strength in another area, while the deficit model rejects this idea and instead suggests using the minimum ability score as the main indicator of a clinically significant deficit in a child’s profile that requires remedial intervention. This minimum ability is operationalized in the AID 3 as the lowest or second-lowest subtest score called the “(lower margin of) intelligence quantity”. Generally, the lowest subtest score would be used, except if there was some apparent reason not to do so, e.g., if it was considered invalid. Then, the second lowest subtest score would be used instead. The authors add the range as the span between the highest and lowest subtest scores to account for the heterogeneity of a test profile. Since practitioners often demand composite scores, the authors also offer the “primary IQ” (P-IQ) as an alternative to an overall IQ score. The P-IQ equals an individual’s factor score of the first factor (“Information Processing of the Societal Environment”) of the factor solution provided in the manual and is thus still in line with the deficit model. However, whether the P-IQ is a practically useful measure in terms of criterion validity has yet to be investigated. It must be noted that according to the authors even the P-IQ can only be considered a “compromise”: Kubinger (2019b) demonstrated with IRT model tests that for both the IQ and P-IQ derived from the AID 3 the models do not hold and the scores are therefore not scientifically sound in terms of IRT. However, when considering that factor analysis results are usually simply assumed to be true, Kubinger (2019b) argues that P-IQ still displays a better choice than an IQ when needing a composite cognitive ability measure. In order to fulfill institutional requirements in certain practical cases, the authors still provide a way to calculate the average of the 12 subtests and convert it into an IQ scale but warn that this score has little scientific basis and should therefore not be used (Kubinger & Holocher-Benetka, 2023). Most subtests in the AID 3 feature adaptive testing, realized in both branched and—with the additional software “AID_3_tailored” (Spohn, 2017)—tailored testing.

*Internal Validity.* The authors report that the AID 3 does not follow any contemporary intelligence theory or model (Kubinger & Holocher-Benetka, 2023). Instead, they offer three different models: They divide the subtests into verbal-acoustic vs. manual-visual abilities, they derive a four-factor model from a principal component analysis (PCA) and offer a model of specific learning disorders. The four-factor PCA model is referenced with a similar, but not very well-known model by E. Roth et al. (1980). As mentioned above, the individual factor score of the first factor in this factor solution is referred to as the P-IQ. The model of specific learning disorders was derived with reference to the author’s goal to use the AID 3 as a screening tool for specific learning disorders. It differentiates between the three factors “Perception”, “Memory”, and “Usage” (Kubinger & Holocher-Benetka, 2023). All three models with their respective subtests can be viewed in detail in Supplementary Materials S5.

#### 1.4.3. Previous Comparative Studies

As Mickley and Renner (2019) summarized aptly, comparing intelligence tests (in the German-speaking area) is a difficult undertaking due to the test batteries’ different focal points, tasks and instructions, subtests and composite scores, and connection to intelligence theories. The same was found to be true in previous descriptive and empirical comparative studies including different editions of WISC (and other Wechsler scales) and AID. Descriptively, intelligence test batteries can be compared by relating the test batteries with their respective subtests to one common intelligence theory, as done by Mickley and Renner (2019) with reference to CHC theory, or Süß and Beauducel (2011) with reference to the Berliner Intelligenzstrukturmodell [Berlin Intelligence Structure Model] (BIS; Jäger, 1982). Empirically, IQ measures from both test batteries can be compared, as done by Schlagheck and Petermann (2006) for the AID 2 and the WISC-III. For reference, the results of these comparisons can be found in Supplementary Materials S4. However, there are no existing empirical studies comparing the AID 3 and the WISC-V, the most recent editions of both test batteries. One reason may have been found by Ziegler and Reichert (2017) who argued that the comparison of the AID 3 to other intelligence tests is difficult because of its explicit distinction from other intelligence structure theories and the *g*-factor. Apart from the AID 3, a large number of studies have aimed at empirically comparing different intelligence test batteries, including comparisons with WISCs. For example, Hagmann-von Arx et al. (2018) were able to show that while different intelligence test batteries (including the WISC-IV) were similar in the sample mean, they showed significant differences on an individual level in 12 to 38% of all cases.

### 1.5. Current Study

This study particularly aims to address a significant gap in the empirical literature concerning the assessment of giftedness in individuals with high mathematical ability. Therefore, a sample is used that was identified as mathematically highly gifted and that relied on an entering test in the framework of a foster program (Kießwetter, 1985). Despite the widespread use of intelligence tests such as the WISC and AID for identifying giftedness in children and adolescents, there is a notable scarcity of research examining how these instruments profile children with exceptional talent in mathematics, especially within German samples (Pauls & Daseking, 2021). Recent research highlights the importance of multidimensional assessment approaches for accurately capturing the heterogeneity of giftedness (Pfeiffer, 2020). Moreover, the current literature presents inconsistent results regarding the factor structure of the WISC, with some studies supporting alternative models such as a four-factor or bifactor solution (Canivez et al., 2021; de Jong, 2023; Pauls & Daseking, 2021). Despite these findings, little is known about the ability profiles of highly mathematically gifted children when assessed with different tools. This study seeks to fill this gap by empirically comparing the ability profiles derived from the WISC-V and the AID 3 in a sample of highly mathematically gifted children and adolescents, thus providing valuable insights into the diagnostic utility of these tests within this specific population.

## 2. Materials and Methods

The data collection was led by Author 2 and her colleague Frank Spohn from the University of Hamburg. The data was collected from November 2019 to January 2020. This study was preregistered to the Open Science Framework on 17 January 2024.

### 2.1. Participants

The participants were 36 mathematically highly talented children and adolescents (25% female, 75% male) aged 12.08 to 14.09 years (*M* = 12.89, *SD* = 0.58) at the point of their first testing date. The participants were recruited from the William Stern Association e.V. which offers a talent promotion program in mathematics. The participants in the program are characterized by extraordinary mathematical abilities and having fun when thinking and solving complex tasks (Hansen et al., 2022; Krüger et al., 2018). Those participants of the program who did not exceed the age required by the standardization of the test batteries (6;0 to 15;11 years; 90 out of 120 participants in total) were eligible to participate. They were invited by letter and informed about the objective of the data collection and the first 40 expressing interest were included in the study. This decision was primarily driven by logistical constraints, including limited resources and available testing appointments within the study timeframe. Notably, the assessment takes up to 6 h per participant and is therefore limited to time constrains. We acknowledge that this may introduce selection bias, and we discuss this limitation in the later part of the manuscript. In total, 36 adolescents and their parents provided consent and were included in the study; no adolescent terminated their participation before the data collection was complete.

### 2.2. Procedure

All test administrators were graduate students pursuing a master’s degree in psychology, who had completed relevant coursework and received supervised practical training in psychometric assessment procedures to ensure procedural competence. Their proficiency was further reinforced through structured training sessions, including detailed instructions and a dedicated question-and-answer session with Klaus Kubinger, an expert in psychometric testing and adaptive assessment, and author of the AID. A formal pilot study was not implemented, primarily due to logistical limitations; however, the comprehensive training and the involvement of an experienced supervisor aimed to uphold the validity and reliability of data collection. The test administrators individually administered the tests to the participants in a room provided by the university. Each adolescent was tested with the WISC-V and the AID 3 on two different dates. Half of all participants were tested with the AID 3 branched version, and the other half with the tailored version. All (primary) subtests and add-on tests/secondary subtests were completed in both test batteries. The order of tests and the AID 3 testing mode (branched or tailored) were randomized independently; 52.3% completed the WISC-V first, the other 47.7% completed AID 3 first. Exactly half completed AID 3 in the branched version and the other half in the tailored version. An interval of 10 to 14 days between both test batteries was aimed at, but due to organizational constraints, this was not achievable in all cases. The mean testing interval was 13.14 days (*SD* = 9.42, range: 7–56). The test results were analyzed and interpreted by the test administrators and re-checked by the study leaders and a working student. As compensation for study participation, the results were then sent out to the participants and their parents if they desired.

### 2.3. Study Design

This study can be considered as a mixed design with one within-subject factor (the two tests) and one between-subject factor (AID 3 version). For a full visualization of the study design including the randomization, see Supplementary Materials S6.

### 2.4. Statistical Analysis

All statistical analyses were conducted using R Statistical Software (Version 4.3.2; R Core Team, 2023) with RStudio Version 4.5.2 (RStudio Team, 2025). For many descriptive analyses, the psych package (Revelle, 2024) was used. All figures were created using ggplot2 (Wickham, 2016). Tables were created with rempsyc (Thériault, 2023). The correlation plot was created using the corrplot package (Wei & Simko, 2021). If not otherwise specified, an α of 0.05 was applied for all significance tests.

#### 2.4.1. Correlation Matrix

Given the limited sample size (N = 36), the correlation analyses should be regarded as exploratory. Due to the sample size, all correlational findings should be interpreted as descriptive heuristics rather than robust parameter estimates. A correlation matrix of all 35 subtest variables was created to review the overall connectedness of the variables, an α of 0.01 was applied in this case. The procedure will be described in more detail in the respective Section 3.

#### 2.4.2. Methods of Profile Comparisons

In the literature, there is a limited amount of information on how to successfully and reliably conduct profile comparisons of test batteries. In one study, Sauerborn (2015) compared the average standard deviation across all subtests within one test battery with the average standard deviation of another test battery. Sauerborn (2015) considered different reliabilities by comparing each individual’s confidence intervals in a separate analysis. In the current study, the aim was to control for the effect of different reliabilities of the various subtest scores within a profile on their respective true scores (regression toward the mean) before comparing the profiles. Huber (1973) provided a way of cleansing individual subtest scores from the effect of the respective subtest reliability and thus reaching a true score estimate, a so-called τ-equivalent. The general equation for linearly transforming raw values into standardized values was provided by Huber (1973, p. 68):

(1) $$y_{ij}=\frac{x_{ij}-A_{j}}{B_{j}}K+L$$

$x_{ij}$ equals the subtest score of participant i in subtest j; K is the standard deviation that was aimed at by the test constructor and L the respective mean. $A_{j}$ and $B_{j}$ are constants that can be changed based on the goal of the linear transformation. For reaching a τ-transformation, $A_{j}$ would be substituted by $\mu \left(X_{.j}\right)$ and $B_{j}$ by $\sigma \left(X_{.j}\right)\sqrt{p_{jj}}$. Both are sketched as theoretical population parameters and may empirically be substituted by the sample estimators $\bar{x_{\cdot j}}$ and $SD\left(x_{\cdot j}\right)\sqrt{Rel}$, respectively. A final, practically applicable equation would therefore be the following:

(2) $${\hat{v}}_{ij}^{\hat{\tau }}=\frac{x_{ij}-\;\bar{x_{\cdot j}}}{SD\left(x_{\cdot j}\right)\sqrt{Rel}}K+L$$

${\hat{v}}_{ij}^{\hat{\tau }}$ symbols the estimated τ-equivalized subtest score of participant *i*. In this application case, K would be substituted with 3 for the WISC-V and 10 for the AID 3, respectively, and L with 10 for the WISC-V and 50 for the AID 3, respectively.

For an exact derivation of the final equation, see Supplementary Materials S8.

Practically, applying the equation was challenging due to the different approaches to the concept of reliability provided in the manuals, as CTT and IRT offer different perspectives on reliability. In CTT, reliability (*Rel*) is defined as the proportion of true score variance in observed score variance, which results in higher *Rel* for better measurement accuracy (Lord & Novick, 1968). In IRT, however, reliabilities are often provided as standard errors of estimation (SEE) which become smaller with better measurement accuracy (Kubinger, 2003). SEEs represent the IRT equivalent of standard errors of measurement (SEM) in CTT (Kubinger, 2003). To use the derived equation above, CTT’s concept of reliability is needed. In the WISC-V manual, reliabilities are exclusively provided based on CTT and mostly in the reliability concept of *Rel*; however, SEM are also provided. Reliabilities in the AID 3 are exclusively provided based on IRT in terms of SEE. Reliability coefficients based on CTT were only available for previous versions of AID, which exclude some of the newer subtests. To approximate reliability from IRT’s standard error of measurement (SEE), we applied Equation (3), which assumes homoscedasticity of error variance. However, in samples with extreme ability levels, such as gifted individuals, this assumption may not be fully valid, as error variance can vary depending on ability (theta). Therefore, results based on this conversion should be interpreted with caution, and future research should explore local reliability estimates conditioned on ability level.

(3) $$Rel=1-\frac{SEM^{2}}{s^{2}}$$

Both CTT-based SEM and IRT-based SEE can be used for *SEM* in this equation. The standard deviation s of the IRT-based ability parameters can be estimated with the following equation, according to Kubinger (2019a):

(4) $$s=\frac{1}{6}\;\cdot \left(\xi_{max}-\xi_{min}\right)$$

The range $\xi_{max}-\xi_{min}$ can be taken from the standardization sample in the AID 3 manual. In his example, Kubinger (2019a) used the minimum and maximum ability parameters for which t-values can be found in the respective table, so this approach was followed in this study.

No SEEs were provided for the AID 3 subtests 5, 7, and 5c. Reliability coefficients will be estimated for subtests 5 and 7 using retest reliability from the AID 2 (Milanovic, 1998, quoted in Kubinger & Holocher-Benetka, 2023). Add-on test 5c will be excluded from the analysis due to different scaling compared to all other subtests.

For the WISC-V, the CTT-based reliability coefficients *Rel* across all age groups provided in the technical manual were used (Wechsler, 2017).

The above-described approach was conducted for all standardized scores (*M* = 10, *SD* = 3) of the WISC-V and all T-values of the AID 3 (*M* = 50, *SD* = 10). After the τ-transformation, the scores remained in their original unit. To allow for comparison, the WISC-V τ-transformed values were additionally converted into T-values (*M* = 50, *SD* = 10).

With the τ-equivalized values, a profile comparison was aimed at by comparing both standard deviations and ranges across the test batteries. For each individual, standard deviations of τ-equivalized subtest scores were computed across all subtests (*j*) of each test battery. This resulted in individual $SD{\left(WISC\right)}_{i}$ and $SD{\left(AID\right)}_{i}$, and $Range{\left(WISC\right)}_{i}$ and $Range{\left(AID\right)}_{i}$. Two paired-sample *t*-tests were conducted, one for comparing the standard deviations across the two test batteries and one for comparing the respective ranges.

## 3. Results

As expected due to the characteristics of the sample, participants in this study achieved extraordinarily high results in the WISC-V subtests. In the WISC-V, subtest scores are quantified using standardized scores (*M* = 10, *SD* = 3). However, in this study, a mean of 14.27 (*SD* = 2.33) was found when calculated across all the WISC-V subtests. The highest mean was found in the “Arithmetic” (AR) subtest (*M* = 16.61, *SD* = 1.95), notably with more than two standard deviations above the mean of the standardization sample, and the lowest mean in the “Cancellation” (CA) subtest (*M* = 9.36, *SD* = 2.79). The highest subtest score for each participant was AR and “Similarities” (SI) in 16.7% of cases, respectively, and “Matrix Reasoning” (MR) in 13.9% of cases. The highest subtest score of 19 was reached 31 times (5.7% of cases), evenly spread across most subtests. The lowest subtest score for each individual was in CA in 52.7% of cases and “Coding” (CO) in 13.9% of cases. For a full display of the WISC-V subtest descriptive results, see Supplementary Materials S9.

Similar result patterns could be found in the AID 3. The AID 3 standardizes subtest scores in T-values (*M* = 50, *SD* = 10)^2^. The participants in this study achieved a mean of 61.20 (*SD* = 8.88) across all sub- and add-on-tests. The highest mean was found in subtest 3 (“Applied Computation”) with a mean T-value of 72.42 (*SD* = 8.27). Similarly, to the comparable subtest AR in the WISC-V, this is also more than two standard deviations above the mean of the standardization sample. The lowest mean was found in subtest 4 (“Social and Material Sequencing”; *M* = 53.42; *SD* = 12.94). The highest subtest score for each participant was subtest 3 in 44.4% of cases and subtest 12 (“Formal Sequencing”) in 16.7% of cases. The highest possible score of T = 81 was reached 36 times in total (5.6% of cases), most commonly in subtest 3 and add-on test 5a (“Immediately Reproducing—figural/abstract”). The lowest subtest score for each individual was subtest 4 in 33.3% and subtest 2 (“Competence in Realism”) in 16.7%. Supplementary Materials S10 provides a full overview of subtest and add-on test scores in the AID 3.

### 3.1. Composite Scores

Composite scores across the WISC-V and the AID 3 can be categorized into those that follow a compensation approach and are therefore scored in IQ values (*M* = 100, *SD* = 15) and those that do not. The latter only occur in the AID 3 and are the main scores offered and recommended by the test authors (Kubinger & Holocher-Benetka, 2023). As expected, lowest subtest scores (*M_T_* = 43.58, PR = 87.1, *SD_T_* = 7.41) and second lowest subtest scores (*M_T_* = 49.14, PR = 93.3, *SD_T_* = 5.90) showed high results. The full descriptive statistics of composite scores which do follow a compensation approach (WISC-V FSIQ, all indexes of the WISC-V, AID 3 IQ and P-IQ) can be viewed in Table 1. The sample demonstrated an exceptionally high “Quantitative Reasoning Index” (QRI; *M* = 134.72, *SD* = 9.07) that can be considered expected given the sample’s mathematical affinity. Notably, the AID 3 P-IQ (*M* = 119.36, *SD* = 8.68) falls below all the WISC-V composite scores (all *p* < .005)^3^ except the “Processing Speed Index” (PSI; *M* = 114.08, *SD* = 15.71, *p* = .841) and the “Cognitive Proficiency Index” (*M* = 125.56, *SD* = 10.98, *p* = .007). Typically, in gifted samples, the WISC-V FSIQ falls below the “General Ability Index” (GAI; Holocher-Ertl & Seistock, 2019; Krüger et al., 2021; Wechsler, 2017), which interestingly was only apparent in 33% of cases in this sample.

#### 3.1.1. General Composite Score Mean Differences

Additionally, differences in mean general composite scores (WISC-V FSIQ and the AID 3 IQ and P-IQ) were tested. The α-level was Bonferroni-corrected to α = 0.025 instead of α = 0.05 to account for the double-testing of each of the three general composite scores in these *t*-tests (Haynes, 2013). The AID 3 IQ scores were significantly higher (*M* = 128.94, *SD* = 11.32) than the AID 3 P-IQ scores (*M* = 119.36, *SD* = 8.68), *t*(35) = 7.07, *p* < .001, *d* = 1.18. Likewise, the WISC-V FSIQ scores were significantly higher (M = 132.36, SD = 6.87) than the AID 3 primary IQ scores, *t*(35) = −8.32, *p* < .001, *d* = −1.39. Both effect sizes indicate a large effect (*d* > 0.80; Cohen, 1988). Only the WISC-V FSIQ and the AID 3 IQ were not significantly different, *t*(35) = −0.36, *p* = .037, *d* = −0.36.

#### 3.1.2. Correlation Matrix of Composite Scores

The correlation matrix of the composite scores with confidence intervals are shown in Table 2.

### 3.2. Correlation Matrix of All Subtests

**Figure 1:**
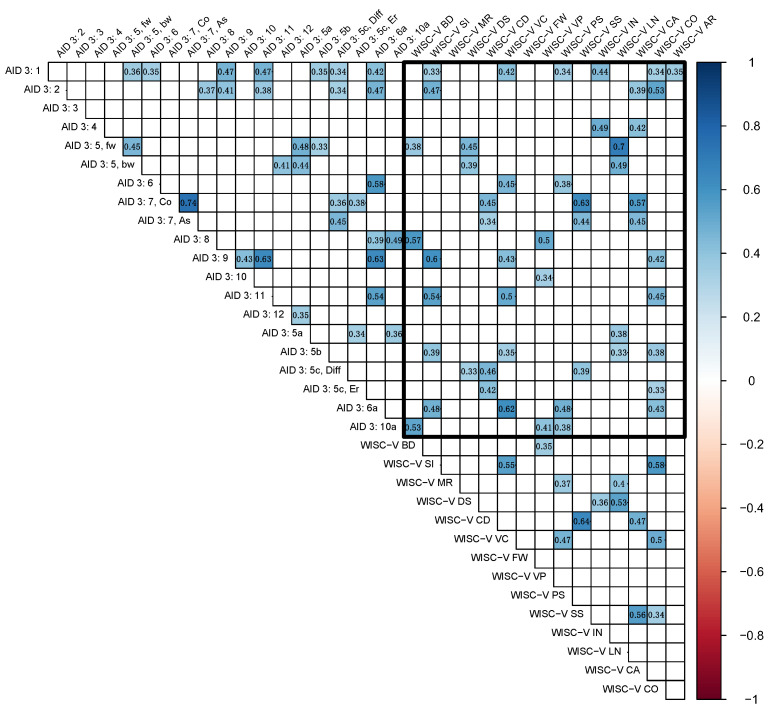
Correlation matrix (α ≤ 0.05), nonsignificant correlations are not displayed. This figure illustrates the correlations (r = −1.0 in red to 1.0 in blue) between the subtests. All AID 3 and WISC-V subtests are indicated from left to right and top to bottom. For a better overview, the central diagonal with r = 1.0 is removed. The black rectangle indicates correlations between the two test batteries, while all correlations outside the rectangle are within one test battery, respectively (AID 3 on the top left, WISC-V on the bottom right). For full subtest and composite score names, please refer to Supplementary Materials S1 and S2, respectively.

The correlation matrix with all significant correlations (α = 0.05) can be viewed in Figure 1.

All subtests except the AID 3 subtest 3 and the WISC-V subtest FW (“Figure Weights”) demonstrated significant correlations (α ≤ 0.05) with some other subtest. Significant correlations were exclusively positive, as is visible in Figure 1.

Within the AID 3 subtests, 16.23% of correlations were significant (31 out of 191). The highest of those were within subtest 7 (“Coding and Associating”), between the two sub-scores “Coding” and “Associating” (*r* = 0.74, *p* < .001), between subtest 9 (“Verbal Abstraction”) and subtest 11 (“Social Understanding and Material Reflection”, *r* = 0.63, *p* < .001) and between subtest 9 and add-on test 6a (“Producing Antonyms”, *r* = 0.63, *p* < .001).

Within the WISC-V subtests, 12.38% of correlations were significant (13 out of 105). The highest of those was between “Coding” (CD) and “Symbol Search” (SS) subtests (*r* = 0.64, *p* < .001).

Between the AID 3 and the WISC-V subtests, $16.\bar{6}$% of all correlations were significant (50 out of 300). The highest correlations were between the AID 3 subtest “Immediately Reproducing—numerical”, sub-score “forward” (5, fw) and the WISC-V subtest “Letter-Number-Sequencing” (LN, *r* = 0.70, *p* < .001), between the AID 3 subtest 7, sub-score “Coding” (7, Co) and the WISC-V subtest SS (*r* = 0.63, *p* < .001), between the AID 3 subtest 9 and the WISC-V subtest SI (*r* = 0.60, *p* < .001), and between the AID 3 add-on test 6a and the WISC-V subtest VC (“Vocabulary”, *r* = 0.62, *p* < .001).

For a detailed display of all correlations including all *p*-values, see Supplementary Materials S14.

**Figure 2:**
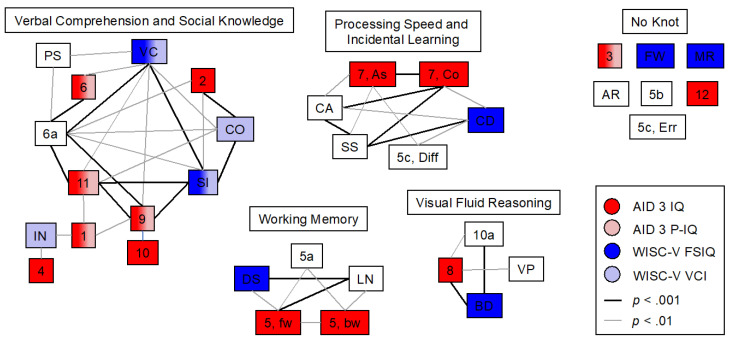
Correlation network (α = 0.01). This figure displays significant correlations between the subtests. Bolder lines indicate a more conservative significance level (α = 0.001), thinner lines indicate a more liberal significance level (α = 0.01). Additionally, overall composite scores are displayed in the respective colors indicated in the legend. For comparative reasons with AID 3 P-IQ, WISC-V VCI was included. The respective knots were named in accordance with factor analyses from the manual, previous studies, and the conducted factor analysis from this study (see Supplementary Materials S13). For full subtest and composite score names, please refer to Supplementary Materials S1 and S2, respectively.

For a more comprehensive overview, a more conservative significance level of α = 0.01 was applied to the correlations and visualized using thicker and thinner lines for stronger and weaker correlations between the subtests. Applying this method, multiple nodes of subtests could be visualized (Figure 2). The four nodes visible in the network were named “Verbal Comprehension and Social Knowledge”, “Processing Speed and Incidental Learning”, “Working Memory”, and “Visual Fluid Reasoning” for better interpretability, based on the factors and index names from the AID 3 and the WISC-V manuals, respectively. All subtests except seven could be allocated specifically to one node. When applying a less strict significance level as in Figure 1 (correlation matrix, α = 0.05) an overall connectedness of the subtests becomes apparent, and the nodes are no longer clearly visible.

### 3.3. Profile Comparisons

The procedure for converting SEMs/SEEs into values conceptually similar to CTT’s reliability coefficient *Rel* was successfully applied for all subtests (except 5c, as elaborated on previously). The derived and used reliability coefficients can be found in Supplementary Materials S11.

Descriptive data of τ-equivalized (and for the WISC-V subtests, additionally, T-transformed) data can be found in Supplementary Materials S12.

Standard deviations and ranges of each participant across all subtests of each test battery were computed. When checking if the differences in standard deviations and ranges, respectively ($SD{\left(WISC\right)}_{i}-SD{\left(AID\right)}_{i}$ and $Range{\left(WISC\right)}_{i}-Range{\left(AID\right)}_{i}$), met the requirements for paired *t*-tests, it became apparent that there was one outlier (<$Q_{1}-1.5\;\cdot IQR$ or >$Q_{3}-1.5\;\cdot IQR$) in the differences in ranges. The outlier was removed for the *t*-test concerning the range only, as it could not be considered an outlier for the difference in standard deviations. The normality assumption still held for the difference in ranges after the removal of the outlier.

**Figure 3:**
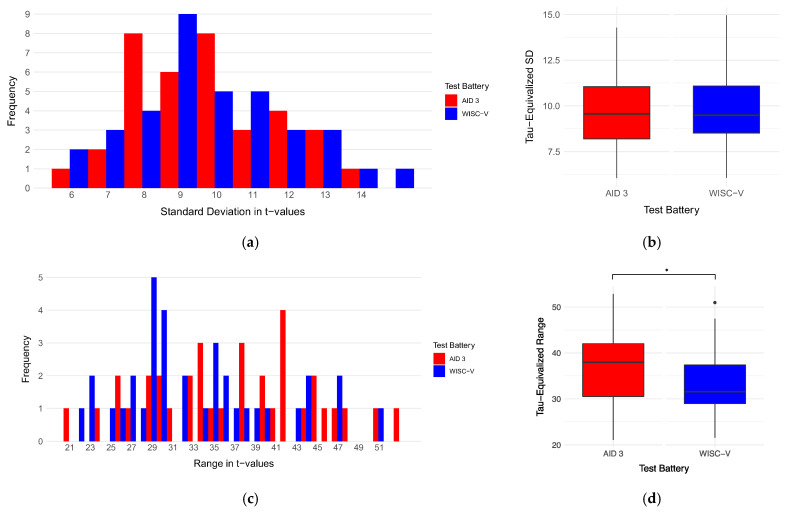
τ-Equivalized standard deviations and ranges. (**a**,**b**) Display the frequencies and distributions of τ-equivalized standard deviations, respectively, for both test batteries, while (**c**,**d**) display the frequencies and distributions of τ-equivalized ranges, respectively, for both test batteries. * means significant difference (*p* ≤ .05) and **·** means outlier.

The τ-equivalized standard deviations of the WISC-V (*M* = 9.80, *SD* = 2.12) and the AID 3 (*M* = 9.75, *SD* = 1.91) were not significantly different, *t*(35) = 0.11, *p* = .91, *d* = 0.02. However, the τ-equivalized ranges of the two test batteries ($M_{WISC}$ = 33.51, $SD_{WISC}$ = 7.39, $M_{AID}$ = 36.94, $SD_{AID}$ = 7.90) were significantly different, *t*(34) = −2.06, *p* = .048, *d* = −0.35. Please note that before the removal of the outlier, the range-*t*-test was not significant. A visual display of the two comparisons (standard deviations and ranges) can be seen in Figure 3.

For exploratory reasons, the *t*-tests were re-run with the original, non-τ-equivalized data. Only the WISC-V subtest scores were converted from their original unit (*M* = 10, *SD* = 3) into T-values (*M* = 50, *SD* = 10) for comparability. Neither standard deviations ($M_{WISC}$ = 9.28, $SD_{WISC}$ = 2.21, $M_{AID}$ = 9.30, $SD_{AID}$ = 2.15) nor ranges ($M_{WISC}$ = 31.57, $SD_{WISC}$ = 8.03, $M_{AID}$ = 33.75, $SD_{AID}$ = 8.56) were significantly different in this case, respectively. However, descriptively, when converting the WISC-V test scores into T-values for comparability^4^, it became apparent that the mean absolute difference in the minimum T-value for each participant ($\left|\min\left(WISC_{i}\right)-\min\left(AID_{i}\right)|\right)$ was 5.95 (*SD* = 4.56, range = 0–19.67). The minimum T-value was lower in the AID 3 than the WISC-V in 63.9% of cases and this difference was significantly different from the expected distribution (50% higher minimum T-value in the AID 3, 50% higher minimum T-value in the WISC-V) across participants (χ^2^(1) = 3.940, *p* < .05). The mean absolute difference in the maximum T-value for each participant ($\left|\max\left(WISC_{i}\right)-\max\left(AID_{i}\right)|\right)$ was 3.15 (*SD* = 3.16, range = 0.33–13) and the maximum T-value was higher in the AID 3 than in the WISC-V in 61.1% of cases; however, this difference was not found to be significant (χ^2^(1) = 2.495).

## 4. Discussion

The sample demonstrated expectedly high subtest and composite scores in both test batteries. While the two IQ values (the WISC-V Full Scale IQ (FSIQ) and the AID 3 IQ) were not significantly different from one another, they were significantly different from the AID 3 Primary IQ (P-IQ), respectively. The correlation network demonstrated visible nodes of subtests when applying a more conservative significance level, where all subtests except seven could be allocated specifically to one node. With a more liberal significance level, the nodes dissolved and hinted at an overarching connectedness of the cognitive abilities tested across the two test batteries. Profile comparisons revealed that while the standard deviations across subtests within each of the two test batteries were not significantly different, ranges were, with AID 3 producing a bigger range than the WISC-V. In the next sections, the results will be discussed with regard to the practical application of these two test batteries.

### 4.1. Main Findings on Composite Scores

The significant difference between the WISC-V FSIQ and the AID 3 primary IQ (*p* < .001) highlights a critical practical implication: the P-IQ, which excludes selected reasoning and mathematical loads, systematically underestimates the child’s overall ability in a sample of high achievers. This suggests that the P-IQ may not be sufficient for capturing the full extent of cognitive capabilities in gifted individuals, and practitioners should interpret it with caution. Given the results of the correlation network (see Figure 2) and the calculation of the composite scores (see Figure 2; for a full display, see Supplementary Materials S5), this cannot be considered surprising. This sample consists of participants with exceptional mathematical abilities and both the WISC-V FSIQ and the AID 3 IQ include several subtests that require mathematical skills. While the AID 3 P-IQ, except for “Applied Computation” (subtest 3), only incorporates subtests that can be considered part of the correlation network “Verbal Comprehension and Social Knowledge”, the mean WISC-V FSIQ and the AID 3 IQ can be expected to reveal higher scores than the AID 3 P-IQ as social knowledge may be an intraindividual weakness in mathematically gifted children. Surprisingly, the AID 3 P-IQ also differed significantly from the WISC-V Verbal Comprehension Index (VCI), again with lower mean scores on the AID P-IQ, even though the respective subtests largely belong to the same node in the correlation network. This difference is smaller than the aforementioned difference to WISC-V FSIQ. One possible explanation for the difference might be the inclusion of the AID 3 subtest 3 in the P-IQ in contrast to the absence of the respective WISC-V subtest “Arithmetic” (AR) in the VCI. However, this provides no explanation as to why the P-IQ was lower than the VCI and not vice versa as expected due to the high scores in AID 3 subtest 3. Again, methodological artifacts may be at play here with low variation in AR leading to a lack of correlations where they would otherwise have been expected. Based on these considerations, this result may not be generalizable to representative samples of the entire child population. Thus, conceptually and empirically, the P-IQ cannot be used as a substitute for a total IQ such as the WISC-V FSIQ (the AID-3 IQ can be used for this purpose). However, the P-IQ does provide valuable information about an individual’s ability in the most relevant area of ability, namely “information processing of the social environment”. In a newer presentation, Kubinger (2019b) suggested using the P-IQ combined with a “secondary IQ”, namely, the factor score of the second factor. These two together, he argues, can then be interpreted in a similar way as “Crystallized Intelligence” and “Fluid Intelligence” (see Horn & Noll, 1997). This approach was incorporated in the AID 3.2 (Kubinger & Holocher-Benetka, 2023). Furthermore, he suggests that the factor scores of the remaining two factors can also be used, and all four factor scores together can provide a sound image of the person’s ability. This approach seems to be very much in line with the WISC-V’s primary indexes, even though the WISC-V does not provide a factor score (a weighted sum) but rather an average of the subtest scores belonging to that factor (a simple sum). The use of factor scores may be a helpful new approach to improving the psychometric quality (see Süß & Beauducel, 2011). However, when implementing new composite scores that are based on factor analyses, it is indispensable to weigh the respective subtests based on their meaningfulness for the factor, thus assigning a weight directly derived from the factor analytic solution. This is not the case in WISC-V as each subtest score is weighed equally in the calculation of the composite scores. The AID 3 provides an overall composite measure for practitioners’ demand. The IQ measure provided in the AID 3 is composed of all AID 3 subtests (excluding add-on tests), representing all four nodes of the correlation network (see Figure 2), similar to the WISC-V FSIQ. The results from this study suggest it would be more appropriate to use the AID 3 IQ instead of the AID 3 P-IQ to compare with the WISC-V FSIQ, at least for mathematically highly gifted children.

### 4.2. Correlation Network and Structure

The discussion of these results should clearly be viewed with caution, as an *α* level of 0.01 was chosen for the study rather than a simple Bonferroni correction (approximately *α* = 0.001 for 35 subtests) or the statistically somewhat more complex false discovery rate (FDR) correction according to the Benjamini–Hochberg method (Benjamini & Hochberg, 1995). This decision was based on the statistical properties of the sample, such as ceiling effects or variance constraints, which lead to lower probabilities of significant coefficients.

The four nodes found in the correlation network were similar to the factors found in the exploratory factor analyses for both the WISC-V (Canivez et al., 2021; Pauls & Daseking, 2021) and AID 3 (Kubinger & Holocher-Benetka, 2023). The factors from the factor analysis (which, however, did not meet the statistical requirements, see Supplementary Materials S13) were also largely overlapping with the nodes. Interestingly, the pairs of subtests AR and subtest 3, as well as “Matrix Reasoning” (MR) and “Formal Sequencing” (subtest 12)—each of which was found to have similar instructions and content (see Supplementary Materials S3)—could not be allocated to any node in the correlation network. These subtests all measure some form of fluid reasoning. Since this study’s sample can be considered exceptionally gifted in this ability, little to no variance can be found here and may be the reason for a lack of correlations. Additionally, FW and add-on tests “Storing by Repetition—lexical” and “Learning and Long-range Memorising—figural/spatial, Errors” (“5b” and “5c, Err”; both AID 3) can be found in “No Node”. Regarding subtest 5c producing exceptionally high values in this sample, a lack of variability is likely a reason for the absence of correlations. Subtest 5b yields relatively low reliability estimates and is tested conventionally (Kubinger & Holocher-Benetka, 2023). Thus, the lack of correlations may be an artifact due to measurement errors. The fact that the AID 3’s “Storing by repetition—lexical” and “Learning and Long-range Memorising—figural/spatial” (add-on tests 5b and 5c) (partly) cannot be found in the WISC-V does not necessarily jeopardize the comparability of both test batteries, as both are only add-on tests and thus only administered if there is a specific reason to do so. Regarding FW, based on observations during the data collection, it may be possible that within this specific sample, this subtest may have measured a unique ability. Participants first have to complete 18 items that only require them to select by simply copying and are then required to switch to logical deduction from item 19 onwards. It is possible that attention processes play a role especially in these first 18 items and that the sudden switch in cognitive demands may be very challenging to some, especially younger, participants. A similar finding was recently reported by Rupp et al. (2025) where 130 gifted children were tested with WISC-V. It is possible that in these gifted samples, FW tests attention in the first items before switching to testing the actual targeted ability—reasoning. For those participants struggling to perform the switch in cognitive demands, FW may not actually test reasoning at all. This would be a compelling argument for a preference for adaptive testing especially with gifted and young participants. Furthermore, the assessment of two distinct abilities, which depends on one’s individual way of task processing, may logically account for the lack of significant correlations with FW. Other subtests that would have been expected to correlate significantly were “Block Design” (BD; WISC-V) and “Analyzing and Synthesizing—abstract” (10; AID 3), and “Picture Span” (PS; WISC-V) and “Immediately Reproducing—figural/abstract” (5a; AID 3), respectively. Especially the former two subtests which share the same instruction, and have similar testing material and targeted ability. As outlined above, a lack of variability may be a technical explanation for this finding. However, the different scoring methods and discontinue criteria should also be considered. While BD includes a maximum of 13 tasks (Wechsler, 2017), subtest 10 includes 30 tasks, but typically, only about six of those would be administered (Kubinger & Holocher-Benetka, 2023). Again, a lack of engagement with items that require less cognitive capacities may lead to a different ability being measured than intended. Instead of the actual targeted ability, the boredom of completing items that are too easy for the participant may lead to a measurement of attention or even personality variables like conscientiousness. This would, again, strongly support the superiority of adaptive over conventional testing in the assessment of highly intelligent individuals. However, the correlation between BD and 10 should be revisited and investigated in future studies. The observation that the four nodes in the correlation network combine to form a single node under less stringent significance levels suggest that both test batteries may be assessing a *g*-factor.

### 4.3. Profile Comparisons and Range

Besides composite scores and individual subtests, test batteries are often empirically investigated based on what range of abilities they cover. A larger standard deviation or range is typically positively interpreted, as presumably a larger spectrum of abilities is covered by the test battery (e.g., Kubinger, 2017a). On the other hand, there are also some researchers currently arguing that tests should rather focus on testing individual abilities instead of a broad range (e.g., Canivez et al., 2021); however, this might be difficult to realize due to practitioners’ economic constraints. Canivez et al. (2021) studied a sample of individuals of average intelligence and raised doubts about the usability of the composite scores supplied with the German WISC-V. Although there were no significant differences in the standard deviations computed across subtests for each participant between the two test batteries, the AID 3 exhibited significantly larger ranges than the WISC-V. Similar results were found in Sauerborn (2015) for her comparison of the AID 3 with the Wechsler Adult Intelligence Scale IV (WAIS-IV) in adults 60 years of age and older. In Sauerborn’s (2015) study, the standard deviations were significantly different with a large effect size, while in this study, only the ranges were significantly different. The fact that the individual participants demonstrated a larger range in the AID 3 suggests that the AID 3 assesses some abilities that WISC-V may fail to assess or under-assess. For most participants, the lowest subtest score they obtained was lower in the AID 3 than in the WISC-V. As reported, the AID 3’s subtests 2 and 4 were lowest for half of all participants. Both subtests do not have resembling subtests in the WISC-V (see Supplementary Materials S3 and S4). Interestingly, subtests 2 and 4 were originally included in AID to resemble WISC’s “Picture Completion” and “Picture Arrangement”, respectively. “Picture Arrangement” was already abandoned in the WISC-IV, while “Picture Completion” was only abandoned with the newest WISC edition. The author’s reason was that the subtest put too much emphasis on the speed component, and that other interesting ability areas were granted more space in the WISC-V (Wechsler, 2017). Both adaptations were not made in the AID 3 and might cause a relevant difference in the two test batteries. These low scores may reflect intraindividual weaknesses in social skills and social interaction in this sample of mathematically gifted youth, when measured visually. However, especially considering that the AID 3 authors suggest using the lowest subtest score (and the second lowest for validation) to judge an overall cognitive ability, having consistency across different test batteries regarding the lowest subtest score is extremely relevant. In this study, the lowest subtest score was on average half a standard deviation lower in AID 3 than in the WISC-V. However, the highest subtest score was also higher in the AID 3 than it was in the WISC-V in the majority of cases. While the respective subtest assessing mathematical ability in each test battery was the highest for each participant in most cases across both test batteries, subtest 12 was the second most common highest subtest score in the AID 3. In sum, these considerations explain the different ranges.

### 4.4. Ceiling Effects

A frequent challenge in intelligence testing at the high ability level is the presence of ceiling effects. (Preckel & Vock, 2021; Süß & Beauducel, 2011). This issue occurred in both test batteries to a similar extent and may be a technical reason for a lack of correlations where they would otherwise be expected. For example, the high mean score (16.6) in the “Arithmetic” subtest suggests a notable ceiling effect, with scores approaching the maximum of 19. This indicates a restricted score variance, which can substantially attenuate the observed correlations between this subtest and others. As a result, the reported correlations are likely downward-biased estimates of the true relationships. Formal correction methods, such as Thorndike’s (Case 2) correction for range restriction, could provide more accurate estimates, but were not applied in this study.

### 4.5. Practical Implications, Strengths and Limitations

This study was the first to conduct a thorough empirical comparison between the AID 3 and the WISC-V. Apart from individuals with specific developmental disorders, highly gifted persons represent one of the most common groups being referred for intelligence testing. Therefore, the sample tested in this study is highly relevant for practice, as individuals with average cognitive ability rarely have cause to undergo intelligence assessment. Additionally, the study was preregistered to limit research bias. Results indicated relevant findings for practice, including the similarity of both test batteries across the subtests and, on the other hand, the difficulty of comparing composite scores that are based on different models.

Future research should also examine certain methodological aspects in greater detail. As Furr (2010) recommends critically questioning the use of double-entry ICC instead of using it as an all-encompassing measure, researchers should analyze the profile elements separately in order to obtain more precise and comprehensible results. It should be noted that ICC offers no clear advantages over simpler, more interpretable methods and can even distort profiles in a way that obscures their meaning. In our study we follow this at least to a certain extent. In addition, studies should be conducted to analyze the underlying Rasch model, as many large-scale assessments use the 2PL model or more complex models that take into account the fact that items may differ in terms of their discriminatory power. This was not possible at this time due to the limitations of the study.

However, some limitations must be considered. Firstly, the sample size is rather small and some minor effects may have remained undetected. Nevertheless, it must be noted, that sample sizes in such extreme ability groups are usually rather small, in the WISC-V, for example, the criterion validity for high ability groups was reported with a sample size of *n* = 21 (Wechsler, 2017). Secondly, the statistical requirements for conducting a factor analysis were not given. Furthermore, we did not collect specific data on participants’ current mathematical performance, which limits the ability to link test profiles directly to math achievement. However, since each participant had successfully completed an entrance exam testing advanced mathematical knowledge and creativity in the past and were successfully part of the talent promotion program during the data collection of this study, it can be assumed that this is a sample of particularly mathematically gifted individuals. A further limitation of this study is the absence of exploratory and confirmatory factor analyses (EFA and CFA) within our sample. Future research should explicitly test the factorial structure in similar samples to further validate the tests’ internal validity. Another key feature of this study, which can be considered both a limitation and a strength, is that the sample consisted solely of highly gifted adolescents, which limits the generalizability of the findings on the convergent and divergent validity of the two test batteries. The ceiling effects typical of such a sample may have influenced the strength and pattern of observed relationships, which may differ in more representative or diverse populations. Caution should therefore be exercised in extending these results beyond similar high-ability groups, and further research with broader samples is necessary to confirm the robustness and applicability of these findings in other populations. A further important limitation stems from the restriction of the sample to the top 1–2% of the ability distribution—a significant range restriction. This ceiling effect reduces the variability in test scores and, consequently, attenuates observed correlations among variables. Such range restriction is a well-known psychometric caveat and should be taken into account when interpreting the results; correlations observed in this highly selected, high-ability sample are probably conservative estimates of the relationships present in the broader adolescent population with more typical ability levels. Nonetheless, the correlation matrix provides valuable insights into the relationships between the variables and suggests possible underlying factors. Furthermore, the test administrators involved in the study were very inexperienced, as they were still psychology students who had little or no prior experience with test administration. Finally, the current results cannot be directly transferred to children and adolescents with average abilities. Thus, the results should be treated with caution and re-investigated in future studies.

## 5. Conclusions

As Schlagheck and Petermann (2006) summarized aptly, intelligence is the field of personality psychology that is most thoroughly researched, but still, there is no common definition on what intelligence is. This leads to the issue that individual results on intelligence partly depend on the test battery (Holocher-Ertl et al., 2006). Most intelligence test batteries have convincing advantages and some have problematic disadvantages, the WISC-V and the AID 3 are no exception. Even though test batteries have their advantages and disadvantages, in practice, one test battery usually has to fulfill all testing needs, as economic constraints take place.

This study demonstrated that, overall, higher IQ scores were obtained with the WISC-V compared to the AID 3. Correlation analyses provided stronger support for the underlying factor structures proposed by Kubinger and Holocher-Benetka (2023) and Canivez et al. (2021) than for the model outlined in the WISC-V manual (Wechsler, 2017). The AID 3 yielded a broader range of individual profiles, which may reflect a more differentiated assessment of cognitive functions.

Mathematical giftedness was not uniformly represented across all composite scores. It was most accurately reflected in the WISC-V FSIQ and QRI, as well as in the AID 3 IQ, and to a lesser extent in the AID 3 P-IQ and WISC-V PSI. As anticipated, subtests involving arithmetic proved to be the most sensitive indicators of mathematical talent. The AID 3 offers methodological and theoretical advantages, particularly in terms of scoring precision and adaptive testing through its foundation in Item Response Theory (IRT). Especially in samples with extreme cognitive abilities, practitioners should consider the superior measurement accuracy of adaptive formats when interpreting test profiles.

Methodologically, some unexpectedly low inter-test correlations emerged, which might be attributable to ceiling effects and reduced score variability within the gifted sample.

In conclusion, this study highlights both considerable overlaps and meaningful differences between the two test batteries. Researchers and practitioners are advised to move beyond a simplistic interpretation of composite scores, particularly when working with highly gifted or heterogeneous profiles. Instead, a more individualized, strength- and weakness-oriented assessment approach is recommended. Future research should continue to explore these discrepancies and their implications for the assessment of cognitive abilities.

## Figures and Tables

**Table 1:** Descriptive data of composite scores in the AID 3 and WISC-V.

Score	Mean	*SD*	Median
AID P-IQ	119.36	8.68	120.00
AID IQ	128.94	11.32	130.00
WISC FSIQ	132.36	6.87	131.00
WISC VCI	127.44	12.52	130.00
WISC VSI	126.61	10.09	127.50
WISC FRI	129.81	8.36	129.50
WISC WMI	127.67	8.15	127.00
WISC PSI	114.08	15.71	114.00
WISC QRI	134.72	9.07	134.50
WISC AWMI	127.44	12.00	126.00
WISC NVI	132.97	8.35	131.00
WISC GAI	131.44	7.23	129.00
WISC CPI	125.56	10.98	125.00

*Note.* Values in IQ values (*M* = 100, *SD* = 15). For full names of composite scores, see Supplementary Materials S1 and S3, respectively.

**Table 2:** Correlation matrix of the composite scores of the AID 3 and WISC-V.

		AID 3		WISC-V										
	Variable	P-IQ	IQ	FSIQ	VCI	VSI	FRI	WMI	PSI	QRI	AWMI	NVI	GAI	CPI
AID 3	Minimum T-Value	0.265 [−0.043; 0.564]	0.597 [−0.337; 0.32]	0.328 [0.246; 0.732]	0.248 [−0.003; 0.591]	0.168 [−0.24; 0.412]	−0.167 [−0.356; 0.3]	0.071 [0.105; 0.657]	0.419 [−0.15; 0.486]	−0.119 [0.157; 0.686]	0.063 [−0.313; 0.344]	0.157 [−0.02; 0.58]	0.22 [−0.302; 0.354]	0.399 [−0.07; 0.546]
	P-IQ		0.699 [0.033; 0.614]	0.289 [0.609; 0.883]	0.698 [−0.068; 0.547]	0.065 [−0.155; 0.483]	−0.2 [0.016; 0.603]	0.298 [−0.43; 0.218]	−0.018 [−0.335; 0.322]	0.004 [0.337; 0.775]	0.188 [−0.44; 0.207]	−0.007 [−0.372; 0.283]	0.371 [0.275; 0.746]	0.121 [0.334; 0.774]
	IQ			0.55 [0.767; 0.935]	0.497 [−0.093; 0.529]	0.39 [0.502; 0.844]	−0.01 [0.037; 0.617]	0.357 [−0.271; 0.384]	0.348 [0.048; 0.623]	0.196 [0.152; 0.683]	0.367 [−0.201; 0.445]	0.392 [0.305; 0.761]	0.485 [−0.253; 0.4]	0.462 [0; 0.593]
WISC-V	FSIQ				0.599 [−0.176; 0.466]	0.458 [0.271; 0.744]	0.521 [0.029; 0.611]	0.648 [−0.181; 0.462]	0.421 [−0.216; 0.433]	0.191 [0.233; 0.726]	0.531 [−0.175; 0.467]	0.781 [0.029; 0.612]	0.875 [0.796; 0.944]	0.657 [−0.087; 0.533]
	VCI					0.136 [0.19; 0.704]	0.035 [0.363; 0.787]	0.306 [−0.117; 0.511]	0.018 [0.27; 0.744]	−0.13 [0.406; 0.805]	0.137 [0.546; 0.86]	0.163 [0.24; 0.729]	0.741 [−0.16; 0.478]	0.158 [−0.17; 0.471]
	VSI						0.116 [0.512; 0.848]	0.326 [0.081; 0.643]	0.267 [0.202; 0.71]	0.243 [0.107; 0.658]	0.162 [−0.179; 0.463]	0.72 [0.332; 0.773]	0.426 [−0.04; 0.566]	0.368 [−0.469; 0.171]
	FRI							0.31 [0.48; 0.835]	−0.05 [0.07; 0.637]	0.576 [−0.147; 0.489]	0.354 [−0.221; 0.428]	0.527 [−0.233; 0.418]	0.595 [−0.085; 0.535]	0.103 [−0.263; 0.391]
	WMI								0.096 [−0.337; 0.32]	0.183 [0.246; 0.732]	0.713 [−0.003; 0.591]	0.551 [−0.24; 0.412]	0.488 [−0.356; 0.3]	0.532 [0.105; 0.657]
	PSI									−0.132 [0.609; 0.883]	0.029 [−0.068; 0.547]	0.554 [−0.155; 0.483]	0.082 [0.016; 0.603]	0.891 [−0.43; 0.218]
	QRI										0.178 [−0.093; 0.529]	0.292 [0.502; 0.844]	0.25 [0.037; 0.617]	−0.031 [−0.271; 0.384]
	AWMI											0.343 [0.271; 0.744]	0.361 [0.029; 0.611]	0.354 [−0.181; 0.462]
	NVI												0.618 [0.363; 0.787]	0.719 [−0.117; 0.511]
	GAI													0.295 [0.081; 0.643]

*Note.* Abbreviations for the composite scores see above.

## Data Availability

The original data presented in the study are available on request in the Open Science Framework at https://osf.io/9h86x (accessed on 12 December 2025).

## Notes

1 The original AID 3 (referred to as the AID 3.1) was published by Kubinger & Holocher-Ertl (2014). With a standardization check (Kubinger & Suster, 2023), the manual and test instructions were revised and published as the “AID 3.2” (Kubinger & Holocher-Benetka, 2023) As the AID 3.2 was not yet available when this study’s data was collected, this current study still uses the so-called AID 3.1. However, as no changes were made to the test battery, the results are fully applicable to the AID 3.2 and the AID 3.2 will be cited and referred to as AID 3 throughout the remainder of this paper.

2 Add-on test 5c is excluded from this rule as only percentages are provided. For this reason, add-on test 5c is not part of the summary statistics here and its descriptive statistics can be found in Supplementary Materials S10.

3 Significance was tested using *t*-tests, or, if the normality assumption was not met, Wilcoxon Signed-Rank Tests were applied, respectively. As AID P-IQ was tested for significance against ten other variables, the Alpha-level was Bonferroni-corrected to α = 0.005.

4 The τ-equivalization was not yet applied.

## Supplementary Materials

The following supporting information can be downloaded at: https://www.mdpi.com/article/10.3390/jintelligence14040052/s1, S1: Translation Between German and English WISC-V Subtest and Composite Score Names; S2: Translation Between German and English AID 3 Subtest and Composite Score Names; S3: Instructions of Subtests in WISC-V and AID 3; S4: Results from Previous Comparative Studies; S5: Composition of Composite Scores in WISC-V and AID 3; S6: Figure Study Design; S7: Variables for Correlation Matrix; S8: *τ*-transformation based on Huber (1973); S9: WISC-V Subtests Descriptive Results; S10: AID 3 Subtests Descriptive Results; S11: Derived Reliability Coefficients for Profile Comparison; S12: Descriptive data of *τ*-transformed data; S13: Factor Analysis; S14: Full display of all correlations (r) including *p*-values; S15: Additional Literature. References (Brown, 2009; de Winter et al., 2009; Hutcheson & Sofroniou, 1999; Kaiser, 1974; Kubinger & Holocher-Ertl, 2014; Moosbrugger & Kelava, 2012; Osborne, 2019; Preckel, 2017; Tabachnick & Fidell, 2014) are in the Supplementary Materials.
