# Supplementary material for: An Exploratory Comparative Study of the Wechsler Intelligence Scale for Children—Fifth Edition (WISC-V) and the Adaptive Intelligence Diagnosticum 3 (AID 3) in a Sample of Mathematically Highly Gifted Children and Adolescents
Source: J Intell. 2026 Mar 26;14(4):52. doi: 10.3390/jintelligence14040052 (PMC13117794; doi:10.3390/jintelligence14040052)
Supplement: Supplementary file 1 [file jintelligence-14-00052-s001.zip › jintelligence-4073693-supplementary.pdf]

Supplementary Material

S 1: Translation Between German and English WISC-V Subtest and Composite Score Names 1

S 2: Translation Between German and English AID 3 Subtest and Composite Score Names 2

S 3: Instructions of Subtests in WISC-V and AID 3 4

S 4: Results from Previous Comparative Studies 6

S 5 Composition of Composite Scores in WISC-V and AID 3 8

S 6: Figure Study Design 11

S 7: Variables for Correlation Matrix 12

S 8: τ-transformation based on Huber (1973) 14

S 9: WISC-V Subtests Descriptive Results 17

S 10: AID 3 Subtests Descriptive Results 18

S 11: Derived Reliability Coefficients for Profile Comparison 19

S12: Descriptive data of τ-transformed data 21

S 13: Factor Analysis 23

S 14: Full display of all correlations (r) including p-values 27

Supplementary Material **S15**: Additional Literature 37

Supplementary Material S1

Translation Between German and English WISC-V Subtest and Composite Score Names

Type	English		German	
	Name	Abb.	Name	Abb.
**Subtests**	Similarities	SI	Gemeinsamkeiten finden	GF
	Vocabulary	VC	Wortschatz-Test	WT
	Information	IN	Allgemeines Wissen	AW
	Comprehension	CO	Allgemeines Verständnis	AV
	Block Design	BD	Mosaik-Test	MT
	Visual Puzzles	VP	Visuelle Puzzles	VP
	Matrix Reasoning	MR	Matrizen-Test	MZ
	Figure Weights	FW	Formenwaage	FW
	Arithmetic	AR	Rechnerisches Denken	RD
	Digit Span	DS	Zahlen nachsprechen	ZN
	Picture Span	PS	Bilderfolgen	BF
	Letter-Number-Sequencing	LN	Buchstaben-Zahlen-Folgen	BZF
	Coding	CD	Zahlen-Symbol-Test	ZST
	Symbol Search	SS	Symbol-Suche	SYS
	Cancellation	CA	Durchstreich-Test	DT
**Composite Scores**	Full Scale Intelligence Quotient	FSIQ	Gesamt-Intelligenzquotient	G-IQ
	Verbal Comprehension	VCI	Sprachverständnis	SV
	Visual Spatial	VSI	Visuell-räumliche Verarbeitung	VRV
	Fluid Reasoning	FRI	Fluides Schlussfolgern	FS
	Working Memory	WMI	Arbeitsgedächtnis	AGD
	Processing Speed	PSI	Verarbeitungsgeschwindigkeit	VG
	Quantitative Reasoning	QRI	Quantitatives Schlussfolgern	QS
	Auditory Working Memory	AWMI	Auditives Arbeitsgedächtnis	AAGD
	Nonverbal	NVI	Nonverbaler Index	NVI
	General Ability	GAI	Allgemeiner Fähigkeitsindex	AFI
	Cognitive Proficiency Index	CPI	Kognitiver Leistungsindex	KLI

Notes. English subtest and composite score names are displayed in the left column, followed by their respective abbreviations. In the right column, the respective German subtest and composite score names with their abbreviations are displayed. Please note that the German WISC-V version only incorporates 15 out of the original 21 U.S. WISC-V subtests. Additionally, the complementary subtests and index scales were not adopted in the German version (Wechsler, 2017).

Supplementary Material S2

Translation Between German and English AID 3 Subtest and Composite Score Names

Type	English name[a]	German name
Subtests	1 Everyday Knowledge	1 Alltagswissen
	2 Competence in Realism	2 Realitätssicherheit
	3 Applied Computation	3 Angewandtes Rechnen
	4 Social and Material Sequencing	4 Soziale und Sachliche Folgerichtigkeit
	5 Immediately Reproducing - numerical	5 Unmittelbares Reproduzieren - numerisch
	6 Producing Synonyms	6 Synonyme finden
	7 Coding and Associating	7 Kodieren und Assoziieren
	8 Anticipating and Combining - figural	8 Antizipieren und Kombinieren - figural
	9 Verbal Abstraction	9 Funktionen abstrahieren
	10 Analyzing and Synthesizing - abstract	10 Analysieren und Synthetisieren - abstrakt
	11 Social Understanding and Material Reflection	11 Soziales Erfassen und Sachliches Reflektieren
	12 Formal Sequencing	12 Formale Folgerichtigkeit
	5a Immediately Reproducing – figural / abstract	5a Unmittelbares Reproduzieren – figural/abstrakt
	5b Storing by Repetition – lexical	5b Einprägen durch Wiederholung - lexikalisch
	5c Learning and Long-range Memorising – figural / spatial	5c Lernen und langfristiges Merken – figural / räumlich
	6a Producing Antonyms	6a Antonyme finden
	10a Recognition of Structures – visuo-motoric	10a Strukturieren - visuomotorisch
Composite/ Summary Scores	(lower margin of) intelligence quantity	(untere Grenze der) Intelligenzquantität
	second lowest subtest score[b]	Zweitniedrigste Untertestleistung
	range of “intelligence”	Range der “Intelligenz”
	primary intelligence quotient (P-IQ)[b]	“Primär”-Intelligenzquotient (P-IQ)
	intelligence quotient (IQ)[b]	Intelligenzquotient (IQ)

Notes. English names of AID 3 subtest and composite/ summary score names are displayed in the left column, their respective German original names are displayed on the right. AID 3 subtests do not have any official abbreviations, instead, the respective numbers are usually used. Subtests are indexed with a number, while add-on tests are indexed with the number of their associated subtest and an indexing letter (e.g., “5b”).

[a] The authors of AID 3 use British English (BE) spelling in their publications. For consistency reasons, American English (AE) spelling is used here.
[b] For these scores, no “official” English translation could be found, so they were translated here.

Supplementary Material S3

Instructions of Subtests in WISC-V and AID 3

WISC-V		AID 3	
Name	Main instruction	Name	Main instruction
SI	Name similarity between two given everyday objects or concepts	9	Name common function between two given objects
VC	Name pictures or visually displayed objects and describe or define given words	6[a]	Name a word with a similar or equal meaning to a given word
		6a[a]	Name the opposite of a given word
IN	Answer general knowledge questions	1	Answer questions on relevant areas of everyday life
CO	Answer questions on general rules or principles of social situations	11	Answer questions on the context of the "societal" environment
BD	Build visually displayed patterns with two-colored cubes within a time limit	10	Build visually displayed patterns with two-colored cubes
VP	Reconstruct a puzzle with three pieces by choosing the correct pieces and putting them together within a time limit		
MR	Complete an incomplete matrix by choosing one alternative	12	Choose the right piece to complete a pattern of respective pieces
FW	Choose the figure out of given figures that balances a scale with different figures out within a given time limit		
AR	Solve small (practical) mathematical problems (visual and verbal tasks)	3	Solve small mathematical problems (visual and verbal tasks)
DS	Repeat a number of digits in the same order, in the reverse order, or in ascending order.	5	Repeat a number of digits in the same order or reverse order
PS	After observing a page in a stimulus book for a specific amount of time, find the respective picture(s) on a new page (if possible in the same order as displayed in the stimulus book)	5a	Pictures in a stimulus book are pointed at in a specific order, the participant has to repeat this pointing in the exact same order
LN	Repeat a number of digits and letters in ascending and alphabetical order		
CD	Assign specific symbols based on a symbol key to stimuli provided on a paper within a given time frame	7 Co[b]	Code symbols based on a symbol key
SS	Find a given target symbol on a page of symbols		
CA	Scan and mark off specific target symbol within a given time frame		

	2	Name missing details on a visual display of an object
	4	Put a number of images into a logical order so they make a story
	7 As[b]	Code symbols from 7 Co from memory without the symbol key
	8	Use given pieces to put them together into a figure
	5b	Reproduce a number of meaningless syllables
	5c	Remember the order of given images and recall them later
	10a	Resolve the different parts of a geometrical pattern by drawing lines on it

Notes. Instructions were derived using the respective manuals. Comparable instructions are displayed side-by-side for a better overview. For the respective full subtest names, please refer to Supplementary Material 1 and 2, respectively.

Supplementary Material S4

Results from Previous Comparative Studies

WISC-V subtests		Corresponding subtests in AID 3 according to *Mickley & Renner (2019)*[a]	*Süß & Beauducel (2011)*[b]	*Schlagheck & Petermann (2006)*[c]
BD		10, 10a, (8)	8, 10, 10a, (2, 4, 5a)	10
SI		n.f.[e]	6, (1, 8, 9, 11)	9
MR		12	(9)	n.a.[f]
DS	fw	5-fw, (5-bw)	(3), 5-fw/bw	5-fw/bw
	bw, s	(5-fw), 5-bw		
CD		7-Co	7-Co/As	n.a.[f]
VC		6, 6a, (1, 11)	6, (1, 8, 11)	6
FW		(12)	n.a.[f]	n.a.[f]
VP		10, 10a, (8)	n.a.[f]	n.a.[f]
PS		n.f.[e]	n.a.[f]	n.a.[f]
SS		(7-Co)	(7)	n.a.[f]
IN		(6, 6a) 1, 11	1, (6, 8, 11)	1
LN		(5-fw), 5-bw	(3), 5-fw/bw	n.a.[f]
CA		(7-Co)	n.a.[f]	7-Co/As
CO		(6, 6a) 1, 11	1, (6, 8, 11)	11
AR		n.f.[e]	2, (3)	3
n.c.[d]		7-As, 5c		2, 4, 8
n.f.[e]		2, 3, 4, 5a, 5b, 9		
n.a.[f]			5c, 6a, 12	12, 5a, 5b, 5c, 6a, 10a

Notes. AID 3 subtests that are not put in brackets can be directly compared to the respective WISC-V subtests, those in brackets can be put in relation with the respective WISC-V subtests without measuring the exact same construct. For the respective full subtest names, please refer to Supplementary Material 1 and 2, respectively.

[a] This study compared AID 3 and WISC-V subtests based on CHC factors.

[b] This study compared multiple intelligence tests in the German intelligence testing realm based on multiple intelligence theories, including the second edition of AID (AID 2) and a prior version of WAIS (Wechsler Intelligence Scale for Adults). Because of subtest similarity, the comparison was still used here wherever applicable. For consistency reasons, only comparisons based on CHC theory will be considered here.

[c] This study compared what proportion of a sample would be assessed as gifted (IQ ≥ 130) in AID 2 and WISC-III [HAWIK-III]. Similarly as for Süß & Beaucudel (2011), information will still be used wherever applicable. Subtests were compared based on descriptions and assumed tested ability of subtests in the respective manuals.

[d] not connectable: These AID 3/ WISC-V subtests did not show any correspondence with WISC-V/ AID 3 subtests. In some cases, there were correspondences with subtests from prior versions that were now abandoned. Those are also labelleld as "n.c.".
[e] no factor: No corresponding or multiple CHC factor was/were found, so the subtests were not assigned. Connections to AID 3/WISC-V subtests may or may not be possible.
[f] not applicable: The subtest was not yet available in the version that was assessed by the study authors.

Supplementary Material S5

Composition of Composite Scores in WISC-V and AID 3

Corresponding subtests		Main composite scores			WISC-V Primary and ancillary indexes										AID 3 task types		AID 3 model of specific learning disorders[c]			AID 3 factor analysis[d]		
WISC-V	AID 3	WISC-V FSIQ	AID 3 PIQ	AID 3 IQ	VCI	VSI	FRI	WMI	PSI	QRI	AWMI	NVI	GAI	CPI	verbal-acoustic	manual-visual	Per-cei-ving	Me-mo-rizing	Pro-ces-sing	2	3	4
SI	9	X	X	X	X								X			X			X			
VC	6[a]	X	X	X	X								X		X				X			
	6a[a]														X							
IN	1		X	X	X										X				X			
CO	11		X	X	X										X				X			
BD	10	X		X		X						X	X			X	X			X		
VP						X						X										
MR	12	X		X			X					X	X			X						
FW		X					X			X		X	X									
AR	3		X	X			X			X					X			X				
DS	5	X		X				X			X			X	X			X				X
PS	5a							X						X		X		X				
LN								X			X											

Corresponding subtests		Main composite scores			WISC-V Primary and ancillary indexes										AID 3 task types		AID 3 model of specific learning disorders[c]			AID 3 factor analysis[d]		
WISC-V	AID 3	WISC-V FSIQ	AID 3 PIQ	AID 3 IQ	VCI	VSI	FRI	WMI	PSI	QRI	AWMI	NVI	GAI	CPI	verbal-acoustic	manual-visual	Per-cei-ving	Me-mo-rizing	Pro-ces-sing	2	3	4
CD	7 Co[b]	X		X					X			X		X		X					X	
SS									X					X								
CA									X													
	2			X												X	X					
	4			X												X	X					
	7 As[b]			X												X		X			X	
	8			X											X		X			X		
	5b														X			X				
	5c															X						
	10a															X	X					

Notes. This table shows what subtests the composite scores in both WISC-V and AID 3 are made of and contrasts the subtests of both tests, based on the results from Supplementary Material 3 and 4. From left to right, first the WISC-V subtests are listed, then the respective AID 3 tests. Those subtests that have very similar content and instructions, and that are supposed to measure the same construct were put in one line. Then, the main composite scores of both tests are listed (IQ values). Next, the remaining composite scores from WISC-V are listed. Since AID 3 does not directly offer other composite scores than the (P-)IQ, different models offered by the authors were used here to further categorize the subtests: The task types as mentioned in the manual, the results from a factor analysis showing the best fit for the

model of specific learning disorders (with the three factors “perceiving”, “memorizing”, and “processing”), and the results from the PCA of AID 3. For all allocations, exclusively the decisions of the authors as described in the respective manuals are reported. For the respective full subtest names, please refer to Supplementary Material 1 and 2, respectively.

[a] Both the WISC-V “Vocabulary” (VC) subtest and the AID 3 “Producing Synonyms” / “Antonyms” (6/ 6a) subtests are supposed to test vocabulary of the participant. However, they have different approaches to this: While in WISC-V, objects on images have to be named, in AID 3 the person is supposed to produce synonyms (subtest 6) or antonyms (add-on test 6a) to given words.

[b] Only the coding sub-score of AID 3 subtest “7 Coding and Associating” resembles the WISC-V subtest “Coding”.

[c] This model was tested for AID 2 and therefore excludes the subtests 12, 5c, and 6a.

[d] The first factor derived from this factor analysis equals AID 3 P- IQ and is therefore not re-listed here. The authors interpret the factors as the following: 1 = “Information processing of the societal environment” (P-IQ); 2 = “Information processing of new content”; 3 = “Perception capacity”; 4 = “(Re-)production ability through structuring” (non-official translations). The add-on tests 5a, 5b, 5c, 6a, and 10a were not included in this analysis. The subtests 2, 4, and 12 did not load .6 or higher on any of the factors and were therefore not assigned a specific factor.

Supplementary Material S6

Study Design

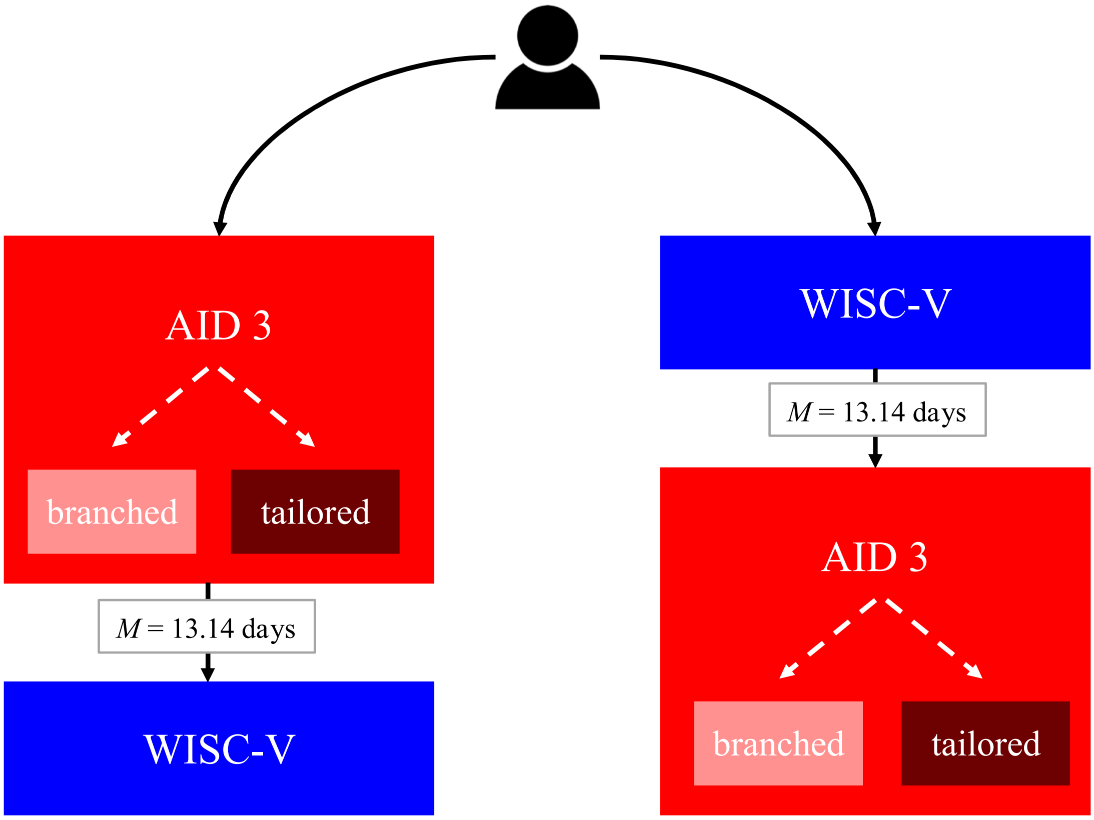

Notes. This figure illustrates the study design. Each participant was randomized into either of the two testing orders (AID 3 first, then WISC-V (left); or vice versa (right)). Additionally, each participant was randomized independently of the first randomization into either of the two AID 3 testing modes (branched or tailored). The mean testing interval between the two testing dates is also displayed.

Supplementary Material S7

Variables for Correlation Matrix

Test battery	Subtest	Variable	Abbre-viation
AID 3	1	T-value in subtest 1	1
	2	T-value in subtest 2	2
	3	T-value in subtest 3	3
	4	T-value in subtest 4	4
	5	T-value in subtest 5 – part “forward”	5, fw
		T-value in subtest 5 – part “backward”	5, bw
	6	T-value in subtest 6	6
	7	T-value in subtest 7 – part “Coding”	7, Co
		T-value in subtest 7 – part “Associating”	7, As
	8	T-value in subtest 8	8
	9	T-value in subtest 9	9
	10	T-value in subtest 10	10
	11	T-value in subtest 11	11
	12	T-value in subtest 12	12
	5a	T-value in add-on test 5a	5a
	5b	T-value in add-on test 5b	5b
	5c	T-value in add-on test 5c – sub-score “Differences”	5c, Diff
		T-value in add-on test 5c – sub-score “Errors”	5c, Er
	6a	T-value in add-on test 6a	6a
	10a	T-value in add-on test 10a	10a
WISC-V	BD	Standardized value[1] in subtest BD	BD
	SI	Standardized value in subtest SI	SI
	MR	Standardized value in subtest MR	MR
	DS	Standardized value in subtest DS	DS
	CD	Standardized value in subtest CD	CD

[1] Standardized value refers to WISC-V standardized values with M=10, SD=3

	VC	Standardized value in subtest VC	VC
	FW	Standardized value in subtest FW	FW
	VP	Standardized value in subtest VP	VP
	PS	Standardized value in subtest PS	PS
	SS	Standardized value in subtest SS	SS
	IN	Standardized value in subtest IN	IN
	LN	Standardized value in subtest LN	LN
	CA	Standardized value in subtest CA	CA
	CO	Standardized value in subtest CO	CO
	AR	Standardized value in subtest AR	AR

Notes. This table shows all variables that were incorporated in the correlation matrix (see Figure 1, 2 and Supplementary Material 14) and the factor analysis (see Supplementary Material 13). The table is divided by test battery (AID 3 in the upper part of the table and WISC-V in the lower part of the table). The subtest abbreviations are displayed on the left (for full subtest names, please refer to Supplementary Material 1 and 2, respectively), followed by the full name of the respective variable and the associated abbreviation as used in the figures and tables for the correlation matrix and factor analysis.

Supplementary Material S8

τ-transformation based on Huber (1973)

This following section describes the derivation of the final equation used for τ-equivalization.

Participant i reached a raw value of x_{ij} of in subtest j. Additionally, the population independent transformation constants *K* and *L* are known. *K* equals the desired standard deviation by the test constructor (3 for subtests of WISC-V, 10 for subtests of AID 3) und *L* equals the desired expectancy value, i.e. *M* (10 for subtests of WISC-V, 50 of subtests of AID 3).

The general transformation of individual raw values into standard values can be reached by applying the following linear transformation:

$$y_{ij} = \frac{x_{ij} - A_j}{B_j} K + L$$

The associated random variable of standard values can (or could theoretically) be derived using the following equation:

$$Y_{ij} = \frac{X_{ij} - A_j}{B_j} K + L$$

The normed true test score is named v_{ij} and equals the expectancy value of Y_{ij}:

$$v_{ij} = \frac{\tau_{ij} - A_j}{B_j} K + L$$

Therefore, the interindividually normed observed variable can be derived in the following way:

$$Y_{.j} = \frac{X_{.j} - A_j}{B_j} K + L$$

According to Huber, for a τ-standardization A_j and B_j would be substituted as follows: $A_j = \mu(X_{.j})$ and $B_j = \sigma(X_{.j})\sqrt{\rho_{jj}}$.

For the τ-standardized random variable Y_{ij}^{τ} the following equation would result:

$$Y_{ij}^{\tau} = \frac{X_{ij} - \mu(X_{.j})}{\sigma(X_{.j})\sqrt{\rho_{jj}}} K + L$$

The τ-standardized true score would therefore (only theoretically) be computable with the expectancy value of the random variable Y_{ij}^{τ}:

$$v_{ij}^{\tau} = \frac{\tau_{ij} - \mu(X_{\cdot j})}{\sigma(X_{\cdot j})\sqrt{\rho_{jj}}}K + L$$

Huber also describes how estimators, which are the only approximation of $\mu(T_{\cdot j})$ and $\sigma(T_{\cdot j})$ in psychological practice, can be used to derive an **inter**individually estimated τ-standardized random variable of standard values:

$$Y_{\cdot j}^{\hat{\tau}} = \frac{X_{\cdot j} - \hat{\mu}(X_{\cdot j})}{\hat{\sigma}(X_{\cdot j})\sqrt{\rho_{jj}}}K + L$$

However, Huber does not provide the "final" equation to standardize **intra**individual raw test values x_{ij} into **intra**individual τ-standardized test values.

Based on the previously described results by Huber, the τ-standardized random variable $Y_{ij}^{\hat{\tau}}$ could be derived using the following equation:

$$Y_{ij}^{\hat{\tau}} = \frac{X_{ij} - \hat{\mu}(X_{\cdot j})}{\hat{\sigma}(X_{\cdot j})\sqrt{\rho_{jj}}}K + L$$

This means, the τ-standardized true score as the expectancy value of $Y_{ij}^{\hat{\tau}}$ could be derived using the following equation:

$$v_{ij}^{\hat{\tau}} = \frac{\hat{\tau}_{ij} - \hat{\mu}(X_{\cdot j})}{\hat{\sigma}(X_{\cdot j})\sqrt{\rho_{jj}}}K + L$$

Still, none of the described components, except for *K* and *L* are practically actually available. The best empirical estimators for the missing components can be considered the following:

For $\hat{\tau}_{ij}$, the raw test value x_{ij} was used, since the true value of the random variable X_{ij} is not available.

For $\hat{\mu}(X_{\cdot j})$, the mean of all $x_{\cdot j}$ in the sample was used.

Similarly, for $\hat{\sigma}(X_{\cdot j})$ the standard deviation (*SD*) of all $x_{\cdot j}$ in the sample was used.

For p_{jj}, already Huber (p. 82) highlights, that problematically reliability parameters are not known *a priori* and have to be determined empirically, as well. The derivation of the reliabilities was elaborated on in the Method section. For a full overview of the derived reliabilities, see Supplementary Material 11.

The final equation for τ-standardizing individual raw values in practice is therefore the following:

$$\hat{v}_{ij}^{\hat{\tau}} = \frac{x_{ij} - \overline{x_{.j}}}{SD(x_{.j})\sqrt{Rel}} K + L$$

with $K = 3$ for WISC-V and $K = 10$ for AID 3, and $L = 10$ for WISC-V and $L = 50$ for AID 3.

Supplementary Material S9

WISC-V Subtests Descriptive Results

Subtest	Mean	*SD*	Median
BD	14.11	1.92	14.00
SI	15.67	2.16	16.00
MR	15.42	1.95	16.00
DS	15.06	1.94	15.00
CD	12.58	2.86	12.00
VC	14.28	2.67	14.00
FW	14.97	1.87	15.00
VP	15.14	2.14	15.00
PS	14.97	1.86	15.00
SS	12.33	3.08	13.00
IN	14.81	2.21	15.00
LN	14.72	2.60	15.00
CA	9.36	2.95	9.00
CO	14.03	2.79	14.00
AR	16.61	1.95	17.00

Notes. Values in standardized values, $M = 10$, $SD = 3$. For the full subtest names, please refer to Supplementary Material 1.

Supplementary Material S10

AID 3 Subtests Descriptive Results

Subtest	Mean	SD	Median
1	60.06	7.41	60.00
2	56.72	12.23	56.00
3	72.42	8.27	73.00
4	53.42	12.94	53.00
5, fw	60.92	10.68	60.50
5, bw	63.81	9.56	64.00
6	58.22	7.13	57.00
7, Co	61.14	11.92	60.00
7, As	57.44	9.78	54.00
8	56.67	9.82	53.00
9	58.89	7.17	60.00
10	64.17	5.17	65.00
11	62.33	8.13	63.00
12	68.11	7.19	69.00
5a	65.56	10.92	62.00
5b	59.25	8.57	60.00
5c, Err	73.24	18.87	87.91
5c, Diff	81.26	17.09	87.91
6a	60.89	8.05	60.00
10a	61.61	4.89	61.00

Notes. Values in t-values with $M = 50$, $SD = 10$. For full subtest names, please refer to Supplementary Material 2.

Supplementary Material S11

Derived Reliability Coefficients for Profile Comparison

Supplementary Material 11a: WISC-V

Subtest	Mean SEM across age groups	*r*
SI	1.06	0.875
VC	1.15	0.853
IN	1.13	0.858
CO	1.25	0.826
BD	1.21	0.837
VP	1.10	0.866
MR	1.15	0.853
FW	0.81	0.927
AR	1.11	0.863
DS	0.89	0.912
PS	1.15	0.853
LN	1.17	0.848
CD	1.19	0.843
SS	1.31	0.809
CA	1.34	0.800

Notes. SEMs for each subtest were derived from the manual, using the mean SEM for all age groups. The respective *r* was derived with the procedure described in the Method section. For full subtest names, please refer to Supplementary Material 1.

Supplementary Material 11b: AID 3

Subtest	ξ_{max}	ξ_{min}	Range	*s*	SEE[b]	*r*
1	7.7	-7.7	15.4	2.57	0.657	0.935
2	5.3	-3.4	8.7	1.40	0.721	0.735
3	8.3	-8.2	16.5	2.75	0.667	0.933
4	7.9	-6.7	14.6	2.43	1.00	0.831
5 fw						0.81[c]
5 bw						0.74[c]

Subtest	ξ_{max}	ξ_{min}	Range	s	SEE[b]	r
6	6.6	-8.8	15.4	2.57	0.670	0.932
7 Co						0.89[c]
7 As						0.64[c]
8	4.8	-6.6	11.4	1.90	0.773	0.834
9	8.5	-6.9	15.4	2.57	0.657	0.935
10	7.5	-10.4	17.9	2.98	1.258	0.822
11	8.1	-7.1	15.2	2.53	0.665	0.931
12	6.5	-6.7	13.2	2.20	0.796	0.869
5a			13.9[a]	2.32	0.970	0.825
5b			13.9[a]	2.32	0.825	0.874
6a	5.8	-6.5	12.2	2.03	0.649	0.898
10a	4.2	-6.2	10.4	1.73	1.024	0.650

Notes. This table illustrates how the final reliability parameters were derived with the procedure described in the Method section. Here, the respective minimum and maximum ability parameters (ξ_{min} and ξ_{max}), the range of those ability parameters, s as described in the Method section, the provided SEE from the manual and the derived r for each subtest are displayed. Add-on test 5c is excluded from this table and the profile analysis since it is scored in percentiles and cannot be compared well to T-values. For full subtest names, please refer to Supplementary Material 2.

[a] No ability parameters were available for this subtest. The range is therefore substituted with the mean of all other ranges, 13.9.

[b] The AID 3 authors report SEEs for every single ability parameter and summarize these by highlighting the minimum and maximum SEE. Here, mean of all SEEs (for the respective age group of the sample) for each subtest was used.

[c] For subtests 5 and 7, no SEEs were provided. Therefore, r is only estimated here based on retest reliability coefficients from Milanovic (1998, as cited in Kubinger & Holocher-Ertl, 2014).

Supplementary Material S12

Descriptive data of τ-transformed data

Test battery	Subtest	Mean	*SD*	Median
WISC-V	BD	50.00	10.69	51.65
	SI	50.00	10.83	48.87
	MR	50.00	10.80	50.95
	DS	50.00	11.00	49.89
	CD	50.00	10.93	49.37
	VC	50.00	10.75	49.30
	FW	50.00	10.83	53.24
	VP	50.00	10.39	50.15
	PS	50.00	10.76	52.15
	SS	50.00	10.47	49.70
	IN	50.00	10.83	50.16
	LN	50.00	10.86	51.16
	CA	50.00	10.89	47.78
	CO	50.00	11.12	52.41
	AR	50.00	11.18	48.63
AID 3	1	50.00	10.34	49.92
	2	50.00	11.66	49.31
	3	50.00	10.35	50.73
	4	50.00	10.97	49.65
	5 FW	50.00	11.11	49.57
	5 BW	50.00	11.62	50.24
	6	50.00	10.36	48.22
	7 Co	50.00	10.60	48.99
	7 As	50.00	12.50	45.60
	8	50.00	10.95	45.91

Test battery	Subtest	Mean	*SD*	Median
	9	50.00	10.34	51.60
	10	50.00	11.03	51.78
	11	50.00	10.36	50.85
	12	50.00	10.73	51.33
	5a	50.00	11.01	46.42
	5b	50.00	10.70	50.94
	6a	50.00	10.55	48.83
	10a	50.00	12.40	48.45

Notes. Values in T-values, $M = 50$, $SD = 10$. WISC-V subtests are displayed in the upper part of the table, AID 3 subtests in the lower part. For full subtest names, please refer to Supplementary Material 1 and 2, respectively.

Supplementary Material S13

Factor Analysis

For WISC-V, CFA were conducted to account for construct validity. In total, 12 models were tested. It was found that at least four factors were necessary to reach an adequate fit based on multiple fit statistics. Two models with four and five factors, respectively, were found to be equally fitting, however, the five-factor model was preferred by the authors due to a better match with the theoretical assumptions (Wechsler, 2017).

Test reviews and studies on WISC-V's construct validity found a multitude of concerns with the way construct validity was assessed and reported in the manual. This included the fact that no EFA were conducted even though major changes in subtests and items would have indicated a necessity to do so (Canivez et al., 2021; Pauls & Daseking, 2021). The construct validity of German WISC-V was revisited by two recent studies (Canivez et al., 2021; Pauls & Daseking, 2021). Both conducted EFA and CFA with similar results. A four-factor solution was found most adequate based on both EFA and CFA. Canivez et al. (2021) found that the factor "Fluid Reasoning" was empirically indistinguishable from *g* (see also Preckel, 2017). They conclude that "it might be necessary to refrain from creating multidimensional measures of intelligence altogether and instead trying to develop multiple unidimensional tests, each designed to measure a single, theoretically well-defined attribute" (Canivez et al., 2021, p. 343). De Jong (2023) came to a similar conclusion using data of 1,000,000 simulated cases in the Dutch version of WISC-V. In his study, both the *g*-factor and the subtest specific factors explained a multiple of the variance that was explained by the broad factors, with some minor exceptions, largely resembling Spearman's original Two-Factor Theory of Intelligence. Overall, it seems reasonable to conclude that the construct validity as reported in the manual must be treated with caution. The results of the studies above overall demonstrate that four factors were more adequate than five and the factors seem to be less robust and meaningful than originally anticipated by the test authors, especially in comparison to the FSIQ.

The described results from factor analyses (for AID 3, please refer to main document) do not hint at specific factors to be expected from this factor analysis,

especially since this exact combination of subtests has not yet been tested. Therefore, exclusively an EFA will be conducted here. The nature of an EFA does not require hypotheses but is rather a hypotheses-generating method (Moosbrugger & Kelava, 2012).

The data was first checked to meet the requirements of factor analyses. For this, the Kaiser-Meyer-Olkin criterion (KMO) was assessed and an overall KMO of > .5 was sought after. Additionally, Bartlett's test for sphericity was conducted to test if the correlation matrix consisted of sufficient correlations to conduct a reasonable factor analysis. All 35 variables that were included in the correlation matrix were also included in the factor analysis (see Supplementary Material 7). With only 36 participants, the cases-per-variable-ratio is nearly 1:1. Recommendations for sample sizes for factor analyses have ranged from 50 to 200 as a minimum sample size (preferably, the sample size would be much bigger), or 3:1 to 20:1 as a minimum cases-per-variable-ratio (de Winter et al., 2009). De Winter et al. (2009) addressed the issue of exploratory factor analyses in small samples and argued that a smaller sample size than the usually recommended minimum of $n = 50$ can be reasonable and yield meaningful results especially if the level of loadings (λ) is high, the number of factors (f) is small, and the number of variables (p) is high. For factor rotation, it is usually suggested to assume correlated factors and apply an oblique rotation method first and proceed to an orthogonal one if factor correlation stays below $r = .32$ (Tabachnick & Fidell, 2014). Specifically, in this case, factors were expected to correlate due to the suspected *g*-factor at a higher level. Different oblique rotation methods (e.g., promax or direct oblimin method) usually do not yield notably different results (see Brown, 2009; Osborne, 2019), so the promax method was used in this case to resemble the studies by Pauls & Daseking (2021) and Canivez et al. (2021). For an orthogonal rotation method, the varimax method was chosen. As suggested in both the previous two studies, salient factor loadings will be considered $\lambda \geq .30$, and a factor will be considered meaningful if at least two variables load saliently on it.

While the data was checked for suitability for EFA, it became apparent that conducting an EFA with this dataset would not yield reasonable results. The overall Kaiser-Meyer-Olkin (KMO) value for factor adequacy was .26, with the absolute minimum usually set to .5 (Hutcheson & Sofroniou, 1999; Kaiser, 1974). Individual

KMO values revealed that only two out of 35 variables demonstrated an acceptable amount (i.e., ≥ .5). In this case, excluding individual variables to raise overall KMO did not seem reasonable or promising. Even though in terms of Bartlett's test of sphericity the data was found to be adequate for factor analysis ($\chi^2(595) = 841.96$, $p < .001$), it was decided that a factor analysis would not be able to yield meaningful results with this dataset.

For demonstrative purposes only, an EFA was still conducted. The results can be seen in Figure 1 of this Supplementary Material (12). The factor analysis revealed that four extracted factors were sufficient. Additionally, correlations between the factors revealed that an oblique rotation was not necessary. The final EFA solution (Figure 1, Supplementary Material 12) therefore consists of four factors and was rotated orthogonally using the varimax method. All factors have seven or more salient loadings. Even when regarding only one test battery, all factors retain sufficient variables with salient loadings to be considered meaningful.

Figure S1 (Supplementary Material S13)

EFA results

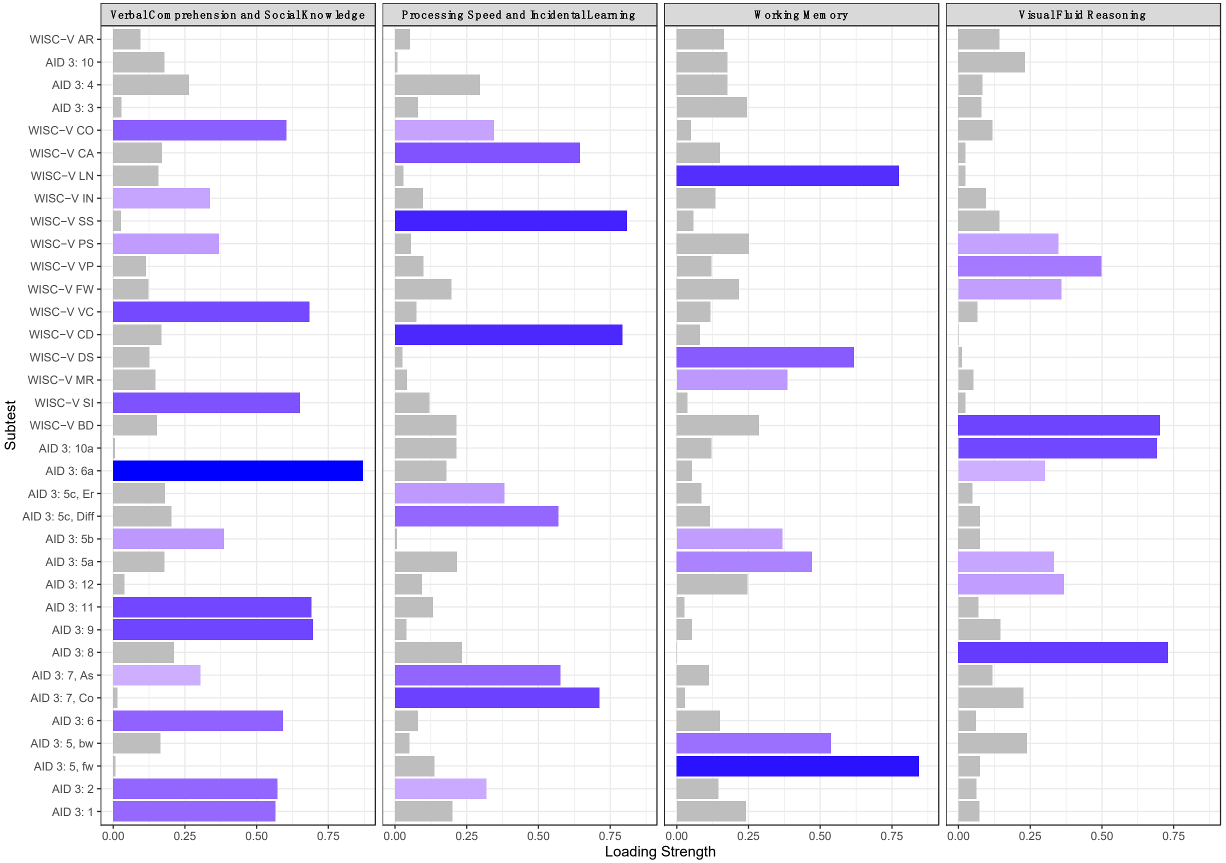

Notes. The figure displays all WISC-V and AID 3 subtests on the left and their respective loadings on the four factors. The factors were named considering previous EFAs and in accordance with the correlation network (Figure 2). All loadings $\boldsymbol{\lambda} < .30$ were not considered meaningful and thus greyed out. For full subtest names, please refer to Appendix 1 and 2, respectively.

Supplementary Material S14

Full display of all correlations (r) including p-*values*

	AID 3: 1		AID 3: 2		AID 3: 3		AID 3: 4		AID 3: 5, fw	
	r	*p*	*r*	*p*	*r*	*p*	*r*	*p*	*r*	*p*
1	1.0		.28	.102	.16	.357	.30	.074	.24	.152
2	.28	.102	1.0		-.18	.291	.28	.097	-.09	.607
3	.16	.357	-.18	.291	1.0		.07	.684	.23	.182
4	.30	.074	.28	.097	.07	.684	1.0		.13	.446
5, fw	.24	.152	-.09	.607	.23	.182	.13	.446	1.0	
5, bw	.36	.034*	.15	.368	.12	.478	.23	.176	.45	.005**
6	.35	.039*	.29	.081	-.11	.537	.21	.213	.14	.418
7, Co	.11	.529	.17	.317	.01	.940	.13	.458	.08	.633
7, As	.23	.174	.31	.065	-.01	.939	.17	.314	-.07	.695
8	.05	.775	.37	.024*	-.07	.683	.13	.441	.10	.580
9	.47	.004**	.41	.013*	.08	.628	.23	.169	.11	.507
10	.26	.123	.04	.831	.11	.526	.08	.641	.32	.054
11	.47	.004**	.38	.023*	-.08	.629	.26	.120	.09	.585
12	-.03	.879	.05	.769	.06	.727	.03	.842	.14	.423
5a	.28	.095	.12	.497	.20	.252	.31	.063	.48	.003**
5b	.35	.035*	.22	.204	.10	.544	.12	.484	.33	.049*
5c, Er	.34	.042*	.34	.044*	-.17	.309	.30	.075	.18	.283
5c, D	.16	.354	.29	.084	.19	.270	.14	.416	.10	.552
6a	.42	.011*	.47	.004**	.11	.523	.22	.196	.01	.961
10a	.02	.928	.09	.591	-.09	.614	.16	.366	-.02	.906
BD	-.11	.520	-.01	.945	.17	.318	.13	.454	.38	.023*
SI	.33	.046*	.47	.003**	-.02	.912	.05	.754	.04	.797
MR	-.08	.647	-.11	.513	.17	.317	.16	.352	.25	.136
DS	.17	.308	.04	.820	.03	.884	.31	.070	.45	.005**
CD	.05	.762	.17	.313	-.02	.896	.19	.255	.16	.353
VC	.42	.012*	.32	.059	.18	.301	.21	.230	.15	.376

	AID 3: 1		AID 3: 2		AID 3: 3		AID 3: 4		AID 3: 5, fw	
	r	*p*	*r*	*p*	*r*	*p*	*r*	*p*	*r*	*p*
FW	-.20	.238	-.23	.175	.29	.090	-.00	.991	.24	.164
VP	.22	.199	-.08	.623	.11	.531	.10	.578	.17	.309
PS	.34	.040*	.04	.812	-.06	.726	.07	.677	.21	.219
SS	.14	.401	.21	.211	-.07	.692	.25	.135	.05	.770
IN	.44	.008**	.15	.393	.07	.705	.49	.002**	.03	.848
LN	.15	.367	-.01	.943	.10	.568	.13	.447	.70	< .001***
CA	.17	.332	.39	.019*	-.09	.613	.42	.012*	.00	.995
CO	.34	.044*	.53	.001***	.05	.793	.07	.668	.03	.854
AR	.35	.035*	.12	.485	.15	.369	.08	.632	.10	.545

	AID 3: 5, bw		AID 3: 6		AID 3: 7, Co		AID 3: 7, As		AID 3: 8	
	r	*p*	*r*	*p*	*r*	*p*	*r*	*p*	*r*	*p*
1	.36	.034*	.35	.039*	.11	.529	.23	.174	.05	.775
2	.15	.368	.29	.081	.17	.317	.31	.065	.37	.024*
3	.12	.478	-.11	.537	.01	.940	-.01	.939	-.07	.683
4	.23	.176	.21	.213	.13	.458	.17	.314	.13	.441
5, fw	.45	.005**	.14	.418	.08	.633	-.07	.695	.10	.580
5, bw	1.0		.11	.511	.18	.290	.02	.922	.27	.106
6	.11	.511	1.0		-.06	.713	.04	.815	.05	.790
7, Co	.18	.290	-.06	.713	1.0		.74	< .001***	.28	.096
7, As	.02	.922	.04	.815	.74	< .001***	1.0		.29	.082
8	.27	.106	.05	.790	.28	.096	.29	.082	1.0	
9	.22	.200	.26	.125	.04	.834	.16	.340	.19	.266
10	.13	.443	.10	.563	.05	.763	-.03	.840	.31	.067
11	.04	.796	.27	.118	-.02	.890	.23	.172	.15	.378
12	.41	.013*	.03	.841	-.10	.564	-.12	.500	.22	.200
5a	.44	.008**	.14	.420	.22	.187	.12	.475	.24	.157
5b	.31	.070	.11	.509	-.03	.867	.19	.258	.10	.576
5c, Er	-.04	.836	.17	.333	.36	.030*	.45	.006**	.16	.337

	AID 3: 5, bw		AID 3: 6		AID 3: 7, Co		AID 3: 7, As		AID 3: 8	
	r	*p*	*r*	*p*	*r*	*p*	*r*	*p*	*r*	*p*
5c, D	.09	.608	.08	.648	.38	.023*	.29	.082	.03	.852
6a	.27	.117	.58	< .001***	-.05	.765	.25	.138	.39	.018*
10a	.02	.887	-.09	.618	.28	.092	.09	.591	.49	.002**
BD	.23	.175	-.20	.238	.32	.060	.15	.385	.57	< .001***
SI	-.06	.717	.30	.074	.09	.600	.27	.113	.17	.335
MR	.13	.438	-.00	.978	-.06	.721	-.20	.237	-.06	.736
DS	.39	.018*	.28	.104	-.02	.913	-.01	.945	.04	.837
CD	.07	.703	-.16	.337	.45	.006**	.34	.045*	.22	.204
VC	.04	.811	.45	.005**	.03	.878	.15	.377	.16	.356
FW	.11	.524	-.01	.963	.08	.661	-.07	.687	.15	.372
VP	.18	.297	.04	.838	.05	.784	-.02	.898	.50	.002**
PS	.17	.316	.38	.021*	.00	.987	.13	.440	.28	.098
SS	-.04	.824	-.06	.742	.63	< .001***	.44	.007**	.26	.123
IN	.18	.292	.32	.055	.18	.294	.23	.170	.07	.705
LN	.49	.003**	.17	.322	.03	.871	.09	.610	.11	.526
CA	.21	.223	.13	.443	.57	< .001***	.45	.006**	.22	.207
CO	-.05	.781	.26	.122	.10	.557	.29	.085	.11	.532
AR	.28	.092	.12	.487	.07	.700	.13	.447	.17	.314

	AID 3: 9		AID 3: 10		AID 3: 11		AID 3: 12		AID 3: 5a	
	r	*p*	*r*	*p*	*r*	*p*	*r*	*p*	*r*	*p*
1	.47	.004**	.26	.123	.47	.004**	-.03	.879	.28	.095
2	.41	.013*	.04	.831	.38	.023*	.05	.769	.12	.497
3	.08	.628	.11	.526	-.08	.629	.06	.727	.20	.252
4	.23	.169	.08	.641	.26	.120	.03	.842	.31	.063
5, fw	.11	.507	.32	.054	.09	.585	.14	.423	.48	.003**
5, bw	.22	.200	.13	.443	.04	.796	.41	.013*	.44	.008**
6	.26	.125	.10	.563	.27	.118	.03	.841	.14	.420
7, Co	.04	.834	.05	.763	-.02	.890	-.10	.564	.22	.187

	AID 3: 9		AID 3: 10		AID 3: 11		AID 3: 12		AID 3: 5a	
	r	*p*	*r*	*p*	*r*	*p*	*r*	*p*	*r*	*p*
7, As	.16	.340	-.03	.840	.23	.172	-.12	.500	.12	.475
8	.19	.266	.31	.067	.15	.378	.22	.200	.24	.157
9	1.0		.43	.009**	.63	< .001***	-.00	.981	.09	.622
10	.43	.009**	1.0		.22	.197	.07	.688	.05	.777
11	.63	< .001***	.22	.197	1.0		-.26	.128	.20	.250
12	-.00	.981	.07	.688	-.26	.128	1.0		.35	.035*
5a	.09	.622	.05	.777	.20	.250	.35	.035*	1.0	
5b	.29	.092	.07	.705	.21	.209	.26	.124	.26	.122
5c, Er	.21	.225	.18	.295	.25	.144	-.04	.836	.10	.562
5c, D	.13	.448	-.15	.393	-.04	.829	-.07	.688	.34	.044*
6a	.63	< .001***	.17	.318	.54	.001***	.10	.549	.27	.108
10a	.16	.342	.11	.537	.03	.879	.14	.410	.36	.030*
BD	.13	.438	.26	.131	-.13	.449	.31	.069	.29	.084
SI	.60	< .001***	.24	.164	.54	.001***	.04	.804	.05	.771
MR	-.16	.363	-.24	.159	-.27	.108	.01	.931	.11	.524
DS	.05	.783	.04	.822	-.06	.732	.17	.329	.27	.105
CD	-.14	.429	-.14	.422	-.03	.859	.01	.977	.24	.162
VC	.43	.009**	.14	.432	.50	.002**	-.09	.604	.20	.253
FW	-.16	.366	.08	.655	-.22	.192	.29	.086	.27	.106
VP	.19	.279	.34	.040*	.13	.443	.29	.086	.29	.086
PS	.22	.196	-.13	.439	.16	.359	.21	.230	.31	.068
SS	.04	.803	.03	.844	.08	.638	-.00	.986	.16	.351
IN	.23	.183	.25	.142	.07	.697	-.07	.699	.09	.598
LN	.17	.327	.13	.454	.10	.555	.16	.355	.38	.023*
CA	.12	.488	-.04	.818	.28	.101	-.11	.509	.14	.401
CO	.42	.011*	.02	.929	.45	.006**	.04	.838	.14	.428
AR	.02	.911	-.03	.861	-.04	.807	.16	.344	.15	.391

	AID 3: 5b		AID 3: 5c, Diff		AID 3: 5c, Er		AID 3: 6a		AID 3: 10a	
	r	*p*	*r*	*p*	*r*	*p*	*r*	*p*	*r*	*p*
1	.35	.035*	.34	.042*	.16	.354	.42	.011*	.02	.928
2	.22	.204	.34	.044*	.29	.084	.47	.004**	.09	.591
3	.10	.544	-.17	.309	.19	.270	.11	.523	-.09	.614
4	.12	.484	.30	.075	.14	.416	.22	.196	.16	.366
5, fw	.33	.049*	.18	.283	.10	.552	.01	.961	-.02	.906
5, bw	.31	.070	-.04	.836	.09	.608	.27	.117	.02	.887
6	.11	.509	.17	.333	.08	.648	.58	< .001***	-.09	.618
7, Co	-.03	.867	.36	.030*	.38	.023*	-.05	.765	.28	.092
7, As	.19	.258	.45	.006**	.29	.082	.25	.138	.09	.591
8	.10	.576	.16	.337	.03	.852	.39	.018*	.49	.002**
9	.29	.092	.21	.225	.13	.448	.63	< .001***	.16	.342
10	.07	.705	.18	.295	-.15	.393	.17	.318	.11	.537
11	.21	.209	.25	.144	-.04	.829	.54	.001***	.03	.879
12	.26	.124	-.04	.836	-.07	.688	.10	.549	.14	.410
5a	.26	.122	.10	.562	.34	.044*	.27	.108	.36	.030*
5b	1.0		.28	.099	.01	.965	.33	.050	.00	.998
5c, Er	.28	.099	1.0		.09	.621	.01	.973	.07	.672
5c, D	.01	.965	.09	.621	1.0		.15	.375	.25	.142
6a	.33	.050	.01	.973	.15	.375	1.0		.14	.427
10a	.00	.998	.07	.672	.25	.142	.14	.427	1.0	
BD	.24	.163	.12	.496	.06	.746	.02	.894	.53	.001***
SI	.39	.018*	.27	.107	.15	.369	.48	.003**	.07	.681
MR	-.10	.573	-.24	.152	.24	.155	-.06	.725	.07	.666
DS	.23	.169	.33	.046*	.14	.402	.18	.300	-.12	.471
CD	-.04	.807	.46	.005**	.42	.012*	-.29	.091	.16	.359
VC	.35	.037*	.13	.438	.31	.067	.62	< .001***	.07	.667
FW	-.11	.517	-.24	.164	.11	.520	.10	.554	.20	.232
VP	.24	.162	.16	.341	-.08	.641	.18	.289	.41	.014*
PS	.25	.142	.04	.829	.14	.411	.48	.003**	.38	.022*

	AID 3: 5b		AID 3: 5c, Diff		AID 3: 5c, Er		AID 3: 6a		AID 3: 10a	
	r	*p*	*r*	*p*	*r*	*p*	*r*	*p*	*r*	*p*
SS	-.20	.252	.39	.017*	.25	.149	-.14	.419	.28	.103
IN	.31	.069	.30	.076	.11	.515	.30	.080	.15	.396
LN	.33	.049*	.09	.614	.06	.715	.22	.207	-.15	.397
CA	-.12	.485	.24	.166	.19	.272	.06	.730	.17	.309
CO	.38	.023*	.25	.144	.33	.050*	.43	.010**	-.08	.649
AR	.07	.702	-.07	.683	-.11	.526	.18	.300	-.11	.526

	WISC-V BD		WISC-V SI		WISC-V MR		WISC-V DS		WISC-V CD	
	r	*p*	*r*	*p*	*r*	*p*	*r*	*p*	*r*	*p*
1	-.11	.520	.33	.046*	-.08	.647	.17	.308	.05	.762
2	-.01	.945	.47	.003**	-.11	.513	.04	.820	.17	.313
3	.17	.318	-.02	.912	.17	.317	.03	.884	-.02	.896
4	.13	.454	.05	.754	.16	.352	.31	.070	.19	.255
5, fw	.38	.023*	.04	.797	.25	.136	.45	.005**	.16	.353
5, bw	.23	.175	-.06	.717	.13	.438	.39	.018*	.07	.703
6	-.20	.238	.30	.074	-.00	.978	.28	.104	-.16	.337
7, Co	.32	.060	.09	.600	-.06	.721	-.02	.913	.45	.006**
7, As	.15	.385	.27	.113	-.20	.237	-.01	.945	.34	.045*
8	.57	< .001***	.17	.335	-.06	.736	.04	.837	.22	.204
9	.13	.438	.60	< .001***	-.16	.363	.05	.783	-.14	.429
10	.26	.131	.24	.164	-.24	.159	.04	.822	-.14	.422
11	-.13	.449	.54	.001***	-.27	.108	-.06	.732	-.03	.859
12	.31	.069	.04	.804	.01	.931	.17	.329	.01	.977
5a	.29	.084	.05	.771	.11	.524	.27	.105	.24	.162
5b	.24	.163	.39	.018*	-.10	.573	.23	.169	-.04	.807
5c, Er	.12	.496	.27	.107	-.24	.152	.33	.046*	.46	.005**
5c, D	.06	.746	.15	.369	.24	.155	.14	.402	.42	.012*
6a	.02	.894	.48	.003**	-.06	.725	.18	.300	-.29	.091
10a	.53	.001***	.07	.681	.07	.666	-.12	.471	.16	.359

	WISC-V BD		WISC-V SI		WISC-V MR		WISC-V DS		WISC-V CD	
	r	*p*	*r*	*p*	*r*	*p*	*r*	*p*	*r*	*p*
BD	1.0		.03	.863	.14	.416	.15	.378	.19	.266
SI	.03	.863	1.0		-.16	.343	.03	.854	-.14	.406
MR	.14	.416	-.16	.343	1.0		.29	.088	.11	.508
DS	.15	.378	.03	.854	.29	.088	1.0		.07	.702
CD	.19	.266	-.14	.406	.11	.508	.07	.702	1.0	
VC	.01	.951	.55	.001***	.21	.223	.14	.414	.01	.945
FW	.17	.330	-.09	.615	.20	.245	.17	.312	-.24	.155
VP	.35	.036*	.13	.437	-.17	.336	.12	.504	.08	.625
PS	.31	.064	.23	.173	.37	.028*	.23	.177	.11	.542
SS	.24	.151	.09	.619	.02	.890	.03	.861	.64	< .001***
IN	.11	.514	.25	.134	.09	.593	.36	.030*	-.08	.658
LN	.21	.228	.08	.624	.40	.017*	.53	.001***	.04	.827
CA	.02	.894	.14	.415	-.13	.462	-.18	.298	.47	.004**
CO	-.12	.475	.58	< .001***	-.19	.277	-.07	.668	.29	.089
AR	.10	.548	-.16	.350	.03	.867	.18	.294	-.07	.681

	WISC-V VC		WISC-V FW		WISC-V VP		WISC-V PS		WISC-V SS	
	r	*p*	*r*	*p*	*r*	*p*	*r*	*p*	*r*	*p*
1	.42	.012*	-.20	.238	.22	.199	.34	.040*	.14	.401
2	.32	.059	-.23	.175	-.08	.623	.04	.812	.21	.211
3	.18	.301	.29	.090	.11	.531	-.06	.726	-.07	.692
4	.21	.230	-.00	.991	.10	.578	.07	.677	.25	.135
5, fw	.15	.376	.24	.164	.17	.309	.21	.219	.05	.770
5, bw	.04	.811	.11	.524	.18	.297	.17	.316	-.04	.824
6	.45	.005**	-.01	.963	.04	.838	.38	.021*	-.06	.742
7, Co	.03	.878	.08	.661	.05	.784	.00	.987	.63	< .001***
7, As	.15	.377	-.07	.687	-.02	.898	.13	.440	.44	.007**
8	.16	.356	.15	.372	.50	.002**	.28	.098	.26	.123
9	.43	.009**	-.16	.366	.19	.279	.22	.196	.04	.803

	WISC-V VC		WISC-V FW		WISC-V VP		WISC-V PS		WISC-V SS	
	r	*p*	*r*	*p*	*r*	*p*	*r*	*p*	*r*	*p*
10	.14	.432	.08	.655	.34	.040*	-.13	.439	.03	.844
11	.50	.002**	-.22	.192	.13	.443	.16	.359	.08	.638
12	-.09	.604	.29	.086	.29	.086	.21	.230	-.00	.986
5a	.20	.253	.27	.106	.29	.086	.31	.068	.16	.351
5b	.35	.037*	-.11	.517	.24	.162	.25	.142	-.20	.252
5c, Er	.13	.438	-.24	.164	.16	.341	.04	.829	.39	.017*
5c, D	.31	.067	.11	.520	-.08	.641	.14	.411	.25	.149
6a	.62	< .001***	.10	.554	.18	.289	.48	.003**	-.14	.419
10a	.07	.667	.20	.232	.41	.014*	.38	.022*	.28	.103
BD	.01	.951	.17	.330	.35	.036*	.31	.064	.24	.151
SI	.55	.001***	-.09	.615	.13	.437	.23	.173	.09	.619
MR	.21	.223	.20	.245	-.17	.336	.37	.028*	.02	.890
DS	.14	.414	.17	.312	.12	.504	.23	.177	.03	.861
CD	.01	.945	-.24	.155	.08	.625	.11	.542	.64	< .001***
VC	1.0		.01	.940	.23	.181	.47	.004**	.07	.677
FW	.01	.940	1.0		.18	.296	-.08	.634	-.03	.849
VP	.23	.181	.18	.296	1.0		.23	.176	.27	.111
PS	.47	.004**	-.08	.634	.23	.176	1.0		.14	.428
SS	.07	.677	-.03	.849	.27	.111	.14	.428	1.0	
IN	.29	.086	.10	.581	.11	.507	.14	.424	.08	.656
LN	.11	.523	.12	.481	.01	.943	.30	.076	.01	.945
CA	.10	.564	-.13	.459	-.12	.481	.05	.778	.56	< .001***
CO	.50	.002**	-.21	.213	.20	.242	.18	.289	.34	.042*
AR	-.16	.351	.08	.630	.14	.426	.12	.503	.02	.898

	WISC-V IN		WISC-V LN		WISC-V CA		WISC-V CO		WISC-V AR	
	r	*p*	*r*	*p*	*r*	*p*	*r*	*p*	*r*	*p*
1	.44	.008**	.15	.367	.17	.332	.34	.044*	.35	.035*
2	.15	.393	-.01	.943	.39	.019*	.53	.001***	.12	.485

	WISC-V IN		WISC-V LN		WISC-V CA		WISC-V CO		WISC-V AR	
	r	*p*	*r*	*p*	*r*	*p*	*r*	*p*	*r*	*p*
3	.07	.705	.10	.568	-.09	.613	.05	.793	.15	.369
4	.49	.002**	.13	.447	.42	.012*	.07	.668	.08	.632
5, fw	.03	.848	.70	< .001***	.00	.995	.03	.854	.10	.545
5, bw	.18	.292	.49	.003**	.21	.223	-.05	.781	.28	.092
6	.32	.055	.17	.322	.13	.443	.26	.122	.12	.487
7, Co	.18	.294	.03	.871	.57	< .001***	.10	.557	.07	.700
7, As	.23	.170	.09	.610	.45	.006**	.29	.085	.13	.447
8	.07	.705	.11	.526	.22	.207	.11	.532	.17	.314
9	.23	.183	.17	.327	.12	.488	.42	.011*	.02	.911
10	.25	.142	.13	.454	-.04	.818	.02	.929	-.03	.861
11	.07	.697	.10	.555	.28	.101	.45	.006**	-.04	.807
12	-.07	.699	.16	.355	-.11	.509	.04	.838	.16	.344
5a	.09	.598	.38	.023*	.14	.401	.14	.428	.15	.391
5b	.31	.069	.33	.049*	-.12	.485	.38	.023*	.07	.702
5c, Er	.30	.076	.09	.614	.24	.166	.25	.144	-.07	.683
5c, D	.11	.515	.06	.715	.19	.272	.33	.050*	-.11	.526
6a	.30	.080	.22	.207	.06	.730	.43	.010**	.18	.300
10a	.15	.396	-.15	.397	.17	.309	-.08	.649	-.11	.526
BD	.11	.514	.21	.228	.02	.894	-.12	.475	.10	.548
SI	.25	.134	.08	.624	.14	.415	.58	< .001***	-.16	.350
MR	.09	.593	.40	.017*	-.13	.462	-.19	.277	.03	.867
DS	.36	.030*	.53	.001***	-.18	.298	-.07	.668	.18	.294
CD	-.08	.658	.04	.827	.47	.004**	.29	.089	-.07	.681
VC	.29	.086	.11	.523	.10	.564	.50	.002**	-.16	.351
FW	.10	.581	.12	.481	-.13	.459	-.21	.213	.08	.630
VP	.11	.507	.01	.943	-.12	.481	.20	.242	.14	.426
PS	.14	.424	.30	.076	.05	.778	.18	.289	.12	.503

	WISC-V IN		WISC-V LN		WISC-V CA		WISC-V CO		WISC-V AR	
	r	*p*	*r*	*p*	*r*	*p*	*r*	*p*	*r*	*p*
SS	.08	.656	.01	.945	.56	< .001***	.34	.042*	.02	.898
IN	1.0		.05	.773	.01	.969	.13	.433	.22	.196
LN	.05	.773	1.0		-.08	.660	.06	.728	.06	.717
CA	.01	.969	-.08	.660	1.0		.18	.296	-.19	.270
CO	.13	.433	.06	.728	.18	.296	1.0		.05	.752
AR	.22	.196	.06	.717	-.19	.270	.05	.752	1.0	

Notes. All correlations (*r*) including their respective *p*-values between all subtests scores are displayed. For a list of included Variables, see Supplementary Material 7. For space reasons, there are separate tables for five variables each and their correlations with all 35 variables. The respective five variables are indicated at the top of the page, whereas the 35 variables are indicated on the left. For full subtest names, please refer to Supplementary Material 1 and 2, respectively. $^{*}p < .05$, $^{**}p < .01$, $^{***}p < .001$

Supplementary Material S15

Additional References

Brown, J. D. (2009). Choosing the right type of rotation in PCA and EFA. *JALT Testing & Evaluation SIG Newsletter*, *13*(3), 20–25.

de Winter, J. C., Dodou, D., & Wieringa, P. A. (2009). Exploratory factor analysis with small sample sizes. *Multivariate Behavioral Research*, *44*(2), 147–181. https://doi.org/10.1080/00273170902794206.

Hutcheson, G., & Sofroniou, N. (1999). *The multivariate social scientist*. Sage. https://doi.org/10.4135/9780857028075.

Kaiser, H. F. (1974). An index of factorial simplicity. *Psychometrika*, *39*, 31–36.

Kubinger, K. D., & Holocher-Ertl, S. (2014). *Adaptives intelligenz diagnostikum 3. Manual* [Adaptive intelligence diagnosticum 3. Manual]. Hogrefe.

Moosbrugger, H., & Kelava, A. (2012). *Testtheorie und Fragebogenkonstruktion* (2. Ed.). Springer.

Osborne, J. W. (2019). What is rotating in exploratory factor analysis? *Practical Assessment, Research, and Evaluation*, *20*(1), 2. https://doi.org/10.7275/hb2g-m060.

Preckel, F. (2017). *Rezension WISC-V—Wechsler intelligence scale for children—Fifth edition*. Karg-Stiftung. Available online: https://www.fachportal-hochbegabung.de/oid/85030/ (accessed on 2 October 2023).

Tabachnick, B. G., & Fidell, L. S. (2014). *Using multivariate statistics* (6th ed.). Pearson.
